# A paradigm shift in cancer research based on integrative multi-omics approaches: glutaminase serves as a pioneering cuproptosis-related gene in pan-cancer

**DOI:** 10.1186/s12905-024-03061-8

**Published:** 2024-04-02

**Authors:** Hai-hong Shi, Joseph Mugaanyi, Changjiang Lu, Yang Li, Jing Huang, Lei Dai

**Affiliations:** 1 Department of Hepato-Pancreato-Biliary Surgery, Ningbo Medical Center Li Huili Hospital, The Affiliated Hospital of Ningbo University, Ningbo, 315040 China; 2 Health Science Center, Ningbo University, Ningbo, 315211 China; 3 Department of Emergency, Ningbo Medical Center Li Huili Hospital, The Affiliated Hospital of Ningbo University, Ningbo, 315040 China

**Keywords:** Glutaminase, Cuproptosis, Pan-cancer analysis, Molecular biomarker, Uterine corpus endometrial carcinoma

## Abstract

**Background:**

Cuproptosis is a newly identified form of unprogrammed cell death. As a pivotal metabolic regulator, glutaminase (GLS) has recently been discovered to be linked to cuproptosis. Despite this discovery, the oncogenic functions and mechanisms of GLS in various cancers are still not fully understood.

**Methods:**

In this study, a comprehensive omics analysis was performed to investigate the differential expression levels, diagnostic and prognostic potential, correlation with tumor immune infiltration, genetic alterations, and drug sensitivity of GLS across multiple malignancies.

**Results:**

Our findings revealed unique expression patterns of GLS across various cancer types and molecular subtypes of carcinomas, underscoring its pivotal role primarily in energy and nutrition metabolism. Additionally, GLS showed remarkable diagnostic and prognostic performance in specific cancers, suggesting its potential as a promising biomarker for cancer detection and prognosis. Furthermore, we focused on uterine corpus endometrial carcinoma (UCEC) and developed a novel prognostic model associated with GLS, indicating a close correlation between GLS and UCEC. Moreover, our exploration into immune infiltration, genetic heterogeneity, tumor stemness, and drug sensitivity provided novel insights and directions for future research and laid the foundation for high-quality verification.

**Conclusion:**

Collectively, our study is the first comprehensive investigation of the biological and clinical significance of GLS in pan-cancer. In our study, GLS was identified as a promising biomarker for UCEC, providing valuable evidence and a potential target for anti-tumor therapy. Overall, our findings shed light on the multifaceted functions of GLS in cancer and offer new avenues for further research.

**Supplementary Information:**

The online version contains supplementary material available at 10.1186/s12905-024-03061-8.

## Highlights

This original study unveiled the biological and clinical connections between the cuproptosis-related gene glutaminase (GLS) and pan-carcinoma.

This study presented an initial endeavor to validate the tumorigenic impact of GLS in pan-cancer.

A comprehensive bio-analysis was conducted to investigate the biological and clinical significance of GLS in uterine corpus endometrial carcinoma.

Identification and prediction of potential drugs and therapies linked to the novel cuproptosis-related gene GLS.

**Supplementary Information:**

The online version contains supplementary material available at 10.1186/s12905-024-03061-8.

## Introduction

With increasing incidence and mortality rates, cancer continues to pose a significant threat to global life expectancy [1, 2]. Although new therapies are being developed, the challenges in cancer treatment persist [3, 4], largely due to the complexity of cancer heterogeneity and the tumor microenvironment [5, 6]. The ten hallmarks used to conceptualize cancer encompass the vast complexity of tumor genotypes and phenotypes: sustaining proliferative signaling, evading growth suppressors, avoiding immune destruction, enabling replicative immortality, tumor-promoting inflammation, activating invasion and metastasis, inducing or accessing vasculature, genome instability and mutation, resisting cell death and deregulating cellular metabolism [7]. Among these hallmarks, cell death mechanisms have been an active area of research, particularly with the discovery of various new patterns such as pyroptosis [8], autophagy [9], and ferroptosis [10]. In light of these findings, investigating the regulatory genes and pathways associated with cell death mechanisms becomes crucial. Furthermore, it is essential to explore their expression, function, and clinical relevance in pan-cancer.

Cuproptosis, a novel cell death pathway [11], is identified with overloaded copper ions binding to lipoylated proteins in the tricarboxylic acid (TCA) cycle, leading to cytotoxic stress [12]. The involvement of the copper-dependent mechanism has been observed in various species and diseases, ranging from antibacterial effect [13] to Wilson’s disease [14] and autophagy in tumorigenesis via reactive oxygen species-dependent CRIP2-APEX2 [15] and AMPK-mTOR pathways [16]. Glutaminase (GLS), as one of the upstream regulators of cuproptosis [11], plays a vital role in regulating metabolism, synthesizing the brain neurotransmitter glutamate, and maintaining acid-base balance in the kidney [17]. Previous researches associated with GLS were mainly focused on neuroinflammation [18], pulmonary hypertension [19] and other metabolic diseases [20]. With the discovery of the tumorigenic role of GLS [21], GLS-driven metabolism pathway alteration was considered contributing to breast cancer progression [22] and pancreatic cancer proliferation [23]. Anna et al. proposed that GLS drove metabolic reprogramming by regulating redox status and autophagy, thereby promoting prostate cancer radiosensitivity [24]. Their findings revealed the potential role of GLS in cancer therapy [25]. However, the role of GLS in pan-cancer through the cuproptosis pathway, along with potential regulatory mechanisms and clinical translation, remains a highly worthwhile exploration frontier.

Motivated by the intriguing nature and substantial potential of GLS, a thorough bioinformatic analysis across pan-cancer was performed in this study. Briefly, GLS expression variances in diverse tissues and carcinomas were explored utilizing extensive public database data, followed by enrichment analysis and clinical relevance assessment of GLS. Furthermore, we investigated the correlation of GLS with tumor immune infiltration, gene mutations, and drug responsiveness. Based on the multi-omics analyses, GLS was determined as a promising molecular biomarker with implications for pan-cancer metabolism and tumor immunology. Additionally, GLS was identified as a potent target tailored for uterine corpus endometrial carcinoma (UCEC).

## Materials and methods

**Fig. 1:**
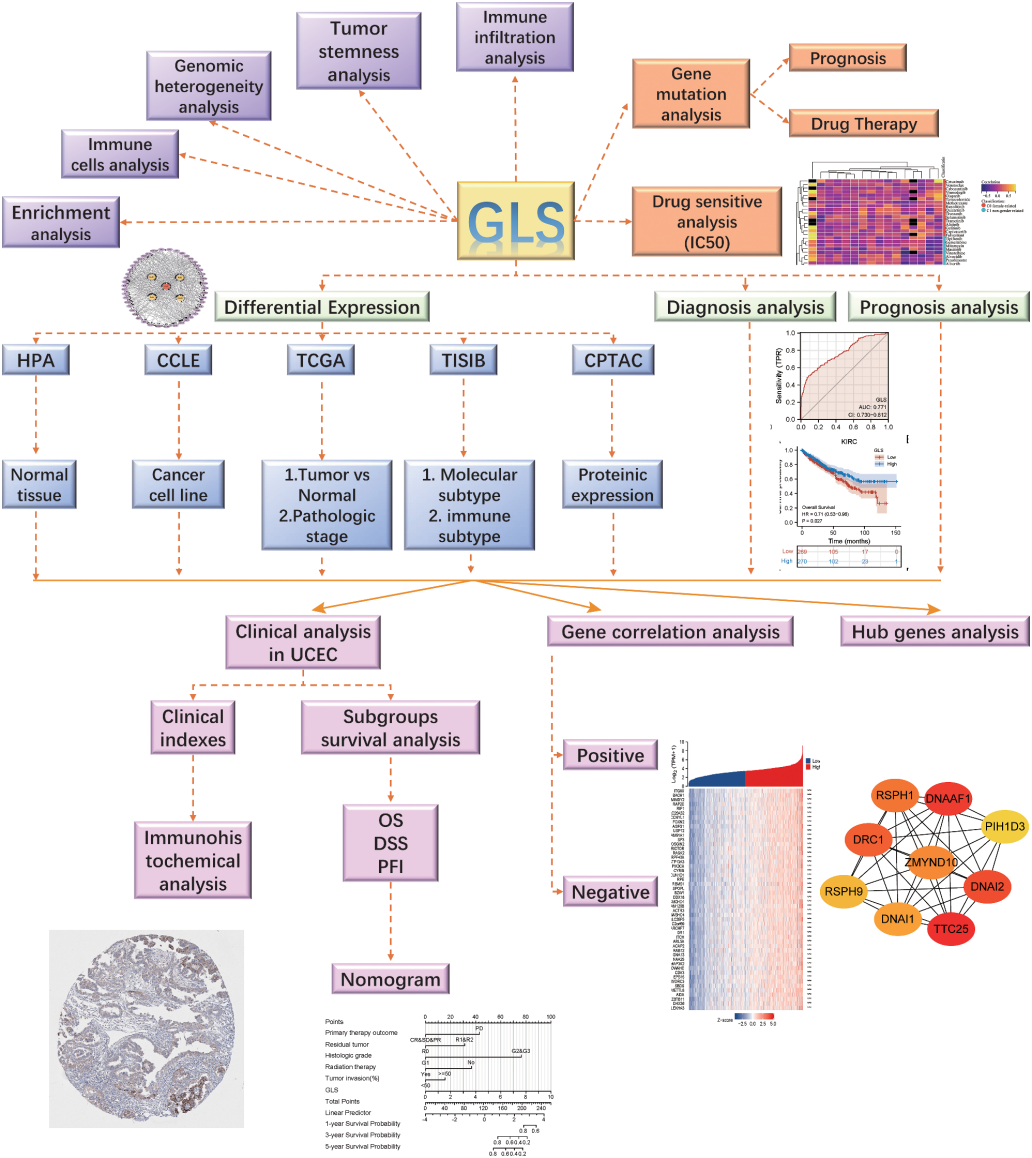
Study design flow chart. TCGA, the cancer genome atlas; UCEC, uterine corpus endometrial carcinoma; GLS, glutaminase; HPA, the human protein atlas; CCLE, cancer cell line encyclopedia; CPTAC, clinical proteomic tumor analysis consortium; OS, overall survival; DSS, disease-specific survival; PFI, progression-free interval

The methodology of this study was presented in the flowchart (Fig. 1).

### Data collection

Pan-Cancer data were searched from the University of California Santa Cruz (UCSC) Xena database (Multi-species Genomic Information Database) (http://genome.ucsc.edu/), including transcriptome data (Transcripts per million reads (TPM) format was selected) and relevant clinical data [26]. Normal tissue data were downloaded from the Human Protein Atlas (HPA) database (integration of proteomic data across various tissues and cell types) (https://www.proteinatlas.org/) [27], and the data from cancer cell lines were acquired from the Cancer Cell Line Encyclopedia (CCLE) database (Encompassing the most comprehensive gene data from human cancer cell lines) (https://sites.broadinstitute.org/ccle/) [28].

### Glutaminase (GLS) expression analysis

Differential expression of GLS was assessed in 27 normal tissues and 30 tumor cell lines. A total of 15,776 tumor and para-cancerous samples from The Cancer Genome Atlas (TCGA) and the Genotype-Tissue Expression (GTEx) database were normalized by Toil procedure [29] and log_2_ transformed for paired/unpaired differential expression analysis.

Then, the GLS expression at different pathological stages across 33 tumors was analyzed via the Gene Expression Profiling Interactive Analysis (GEPIA) database (http://gepia.cancer-pku.cn/index.html) [30]. This database could support large-scale expression profiling and interactive analysis.

Differential expression analysis of GLS at the protein level in pan-cancer was conducted using the University of Alabama at Birmingham Cancer (UALCAN) data analysis portal (http://ualcan.path.uab.edu/analysis-prot.html) [31]. This analysis portal provided proteomic information from the Clinical Proteomic Tumor Analysis Consortium (CPTAC) database (https://cptac-data-portal.georgetown.edu/) [32].​Z-values indicated standard deviations from the median across samples for a given cancer type.

​Additionally, the correlation between GLS expression and molecular or immunological subtypes in various cancers was investigated via the tumor-immune system interaction database (TISIDB) (http://cis.hku.hk/TISIDB/index.php) [33]. This web portal provided a multidimensional analysis of tumor-immune interactions with integrated data.

### Diagnostic capability analysis

Diagnostic capability of GLS in pan-cancer was assessed using receiver operating characteristic (ROC) curves, and a series of analyses were performed based on the TCGA and GTEx database data. The area under the curve (AUC) was calculated for quantifying the diagnostic value. High accuracy was defined as AUC>0.9, medium accuracy was described as 0.7 < AUC ≤ 0.9, and low accuracy was defined as 0.5 < AUC ≤ 0.7 [34].

### Prognostic capability analysis

Survival analysis series on the association between GLS expression and prognosis in pan-cancer was conducted via Kaplan-Meier (K-M) plots. The predictive value was assessed using overall survival (OS), disease-specific survival (DSS) and progression-free interval (PFI). The hazard ratio (HR) and 95% confidence interval (CI) with *p-*value were calculated together.

### Glutaminase (GLS)-related DNA methylation analysis

MethSurv is the first tool to assess DNA methylation biomarkers for prognosis using multivariable survival analysis, enabling cluster analysis of all CpG sites (https://biit.cu.ut.ee/methsurv/) [35]. In this research, MethSurv was utilized to perform survival analysis on GLS DNA methylation levels and specific cancers, aiming to further examine the relationship between gene expression and clinical phenotypes.

### Protein-protein interaction (PPI) network analysis

A total of 50 GLS-related proteins were acquired from the STRING database (http://string-db.org/) [36]. This database provided support for functional proteomic interaction analysis. The main parameters were set as follows: active interaction sources (“Text mining & Experiments & Databases”), max number of interactors displayed [“1st shell: no more than 50 interactors”], minimum required interaction score [“medium confidence (0.400)”] and others (default). Afterward, the Cytoscape (version 3.9.1) [37, 38], an open software for network data integration and visualization, was employed to construct the GLS-related protein-protein interaction (PPI) network.

### Gene enrichment analysis

Kyoto encyclopedia of genes and genomes (KEGG) pathway analysis was conducted, as well as Gene ontology (GO) analysis, for 50 GLS-related proteins using the “tidyr”, “ggplot2” and “clusterProfiler” packages in R (version 4.0.3, www.r-project.org) [39]. Bubble diagrams and circle plots were adopted for visualization.

### Clinical relevance and subgroup survival analysis in uterine corpus endometrial carcinoma (UCEC)

Clinical data from TCGA-UCEC was extracted and cleaned for 552 cases. Patients were divided into two groups based on the GLS expression level (high expression [50–100%] vs. low expression [0–50%]). Baseline features of TCGA-UCEC patients were assessed, followed by a series of analyses on the correlation between GLS expression and various clinical indexes (e.g., histological type, race, weight, etc.).

Corresponding prognostic data [40] were searched for subgroup survival analysis of OS, DSS and PFI. We identified diverse risk factors related to GLS expression and prognosis in UCEC and acquired all the K-M curves using the “survival/survminer” package in R.

### Construction of glutaminase (GLS)-related prognostic model

Based on the above analysis, prognostic values of GLS and clinical features were further evaluated in the OS of UCEC patients by the univariate and multivariate analysis. A GLS-related nomogram involving 5 clinical indicators (primary therapy outcome, residual tumor, histologic grade, radiation therapy and tumor invasion) for 1-, 3-, and 5-year OS probability prediction in UCEC patients was established. Next, relevant prognostic calibration analysis, time-dependent ROC curves and decision curve analysis (DCA) were performed to examine the predictive model validity. Additionally, GLS-related immunohistochemical comparison between UCEC and corresponding normal tissues was obtained from the HPA database [27].

### Glutaminase (GLS)-related gene co-expression analysis in uterine corpus endometrial carcinoma (UCEC)

Subsequently, the positive and negative correlations of the top 50 co-expressed genes in UCEC with GLS expression were investigated. All co-expressed genes, including their Z-score and *p*-value, were presented in the correlation heatmaps. Scatter plots were utilized to exhibit the correlations between GLS and the top six co-expressed genes employing Pearson correlation analysis.

### Glutaminase (GLS)-related differentially expressed genes (DEGs) analysis in uterine corpus endometrial carcinoma (UCEC)

The UCEC samples were classified into two groups based on their GLS expression level ([high expression: 50–100%] vs. [low expression: 0–50%]), followed by the exploration of DEGs between them. The results were visualized via a volcano plot with the threshold values set as follows: [gene biotype: protein-coding; │log_2_ Fold-change (FC)│>1.5 & adjusted *p*-value < 0.05]. GO and KEGG analyses were conducted on the DEGs, and a chord diagram was generated to display relevant pathways of the enrichment analysis, including biological process (BP), molecular function (MF) and cellular component (CC).

In addition, the “multiple proteins” module of STRING [36] was utilized to establish the PPI network of the DEGs set with default parameters, and the GLS-related hub genes in UCEC were explored using 12 algorithms from the CytoHubba module in Cytoscape (version 3.9.1) [38].

### Correlation analysis between glutaminase (GLS) expression and immune cell infiltration

Sangerbox database 3.0 (http://vip.sangerbox.com) [41–43], which integrates multiple databases and processes data in batches, is a user-friendly bioinformatic platform that provides numerous interactive analyses. The potential relationships between GLS expression and immune cell infiltration in pan-cancer were evaluated using this platform. The parameters were established as follows: [data source: TCGA; data transformation: log_2_ (x + 1); samples with the expression level of 0: filter out]. The expression profile was mapped to Gene Symbol to assess the 22 immune cell infiltration scores for each patient in each carcinoma utilizing CIBERSORT algorithms [44] in the “IOBR” package (version 0.99.9) of R [45]. Then, Pearson’s correlation coefficients were calculated using the “psych” package (version 2.1.6) in R, and a heatmap was recruited to visualize the overall analysis, with scatter plots displaying the five most associated cancer types.

### Correlation analysis between genomic heterogeneity and glutaminase (GLS) expression

The accumulation of some mutations in tumor cell proliferation can promote tumor evolution in space and time, resulting in the birth of tumor cell subpopulations carrying specific sub-clonal mutations known as intra-tumor heterogeneity (ITH) [46, 47]. This is closely associated with drug resistance in cancer treatment, which greatly limits the effectiveness of cancer therapy. As a result, we examined the correlation between GLS and three significant ITH indicators in pan-cancer, namely “Tumor Mutation Burden” (TMB) [48], “Microsatellite Instability” (MSI) [49] and “Homologous Recombination Deficiency” (HRD) [50]. The simple nucleotide variation dataset was acquired from TCGA [51], and TMB/MSI/HRD values for each cancer were calculated using the Sangerbox database [41] with the same established parameters. Next, the correlations with GLS were tested using the Pearson method and visualized by a histogram.

### Correlation analysis between tumor stemness and glutaminase (GLS) expression

Stem cell-like tumor phenotype, another distinguishing characteristic of cancer, is bound up with proliferation, metastasis and drug resistance [52]. Hence, two tumor stemness indices, the RNA-based stemness score (RNAss) and the DNA methylation-based stemness score (DNAss), were searched from the previous study [53] to explore their relationships with GLS in pan-cancer. The strength of these associations was tested using spearman’s rank correlation test [54] via the Sangerbox web tool.

### Glutaminase (GLS)-related tumor-immune infiltration analysis

Further, the correlation between GLS expression and tumor-immune infiltration level in pan-cancer was assessed with the assistance of the Sangerbox database. The immune score was calculated by the “ESTIMATE” package (version 1.0.13) [55] in R, and Pearson’s test was adopted for statistical analysis.

### Genetic alteration analysis

We selected 32 TCGA PanCancer Atlas studies (10,967 samples) for genetic alteration visualization and analysis of GLS via the cBioPortal database (http://www.cbioportal.org) [56–58]. The alteration frequency, mutation type and copy number alteration (CNA) associated with GLS in pan-cancer were extracted and summarized. Then, we obtained a pattern diagram showing different mutation types, sites, corresponding protein changes, and cancer types. GLS alteration-related survival analysis (OS, disease-free survival [DFS], DSS and progression-free survival [PFS]) in UCEC was performed using the log-rank test.

### Drug sensitivity analysis

We predicted that BPTES (GLS inhibitor) was the only drug potentially targeting GLS using TISIDB [33], which integrated the data from the DrugBank database [59]. Next, the half-maximal inhibitory concentration (IC50) distribution of BPTES by tissue type in pan-cancer was explored via the Genomics of Drug Sensitivity in Cancer (GDSC) database (http://www.cancerRxgene.org) [60]. After integrating drug response data and genomic markers of sensitivity by this web-portal, a correlation analysis between genomic markers and BPTES-related drug sensitivity was conducted using ANOVA. Besides, BPTES IC50 values between genomic markers and wild type (WT) were also compared in different tumor cell lines.

PharmacoDB (https://pharmacodb.ca/) [61], supporting mining multiple cancer pharmacogenomic datasets publicly, was involved in the evaluation of the association between top anti-tumor compound response and GLS expression in pan-cancer. Upon eliminating the duplicate/missing data, 57 clinically common anti-tumor agents of the GDSC1 dataset were included for correlation analysis.

### Statistical analysis

All statistical analyses and plots were performed with R (3.6.3). For normally distributed variables, T-test was used fro comparison between two groups and one-way ANOVA tests was applied to compare among multiple groups, while nonparametric tests were applied to non-normally distributed variables. Survival analyses were conducted using the Log-rank test or Cox regression test, and correlation analyses were performed using Pearson’s or Spearman’s rank correlation test. Statistically significant was defined as follows: *p* < 0.05, *; *p* < 0.01, **; *p* < 0.001, ***. The correlation coefficient r was defined as follows: 0 < IrI < 0.3: weak; 0.3 ≤ IrI < 0.5: moderate; 0.5 ≤ IrI < 0.7: strong; 0.7 ≤ IrI ≤ 1.0: very strong [62].

## Results

### Differential expression analysis of glutaminase (GLS) in pan-cancer

**Fig. 2:**
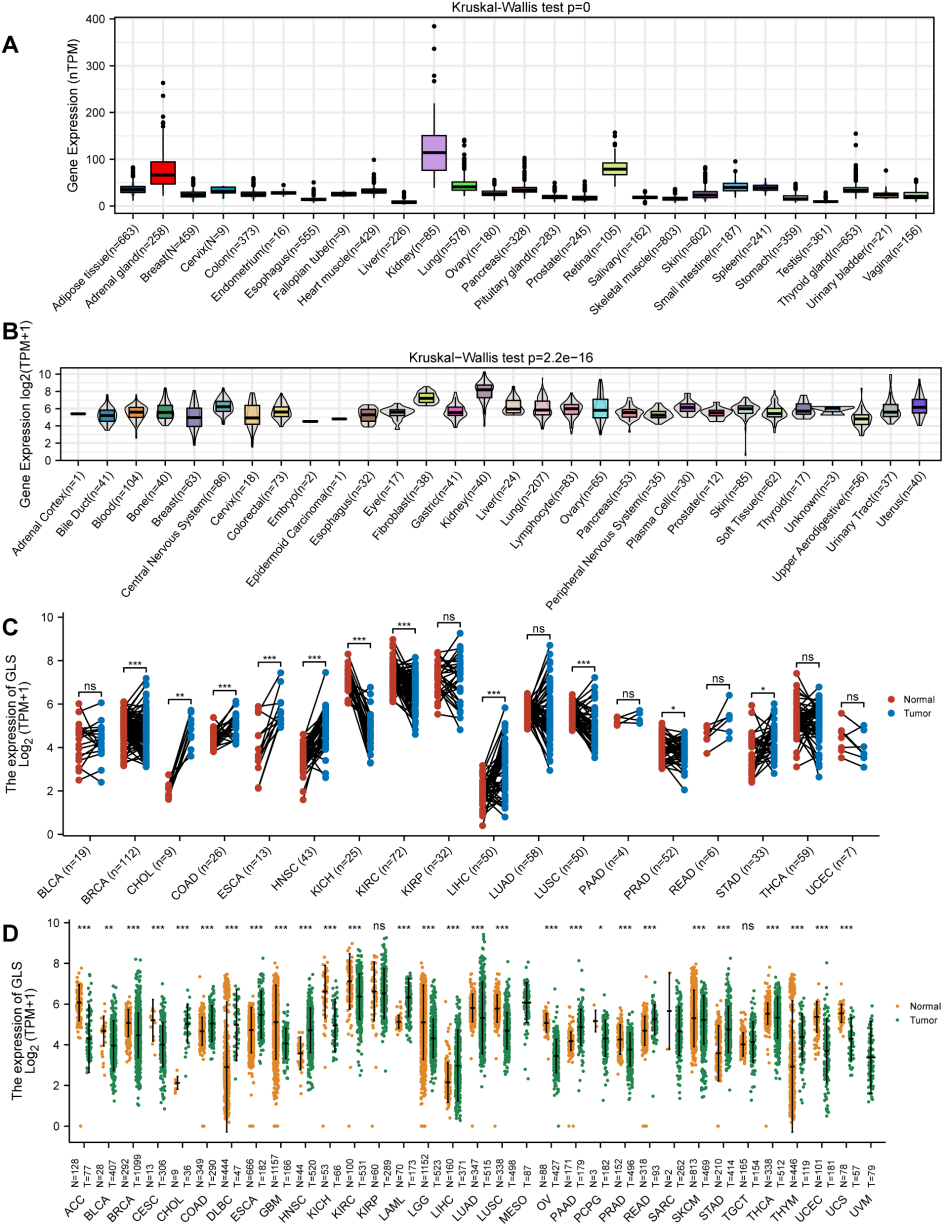
Comparative analysis of glutaminase (GLS) gene expression between tumors and normal tissues. (**A**) Comparison of GLS expression in normal tissues; (**B**) Comparison of GLS expression in cancer cell lines; (**C**) Paired comparison of GLS expression level between TCGA tumors and adjacent normal tissues; (**D**) Unpaired comparison of GLS expression level between TCGA tumors and normal tissues using GTEx data as controls. GLS, glutaminase; TCGA, the cancer genome atlas; GTEx, genotype-tissue expression. *P < 0.05, **P < 0.01, ***P < 0.001

We compared GLS expression levels in 27 normal tissues from the HPA database and discovered that GLS expression was low across most normal tissues. By comparison, the top 3 highest expressed tissues were kidney, adrenal gland and retina (*p* < 0.001) (Fig. 2A). On the contrary, GLS expression was increased in almost all cancer cell lines, and the highest expressed tissue was the kidney (*p* = 2.2e-16) (Fig. 2B). Then, Wilcoxon signed rank test was performed on matched pairs samples from TCGA pan-cancer. The observation results revealed that GLS expression was significantly up-regulated in six carcinoma types, including colon adenocarcinoma (COAD), head and neck squamous cell carcinoma (HNSC), liver hepatocellular carcinoma (LIHC) (all *p* < 0.001), cholangiocarcinoma (CHOL) (*p* = 0.004), esophageal carcinoma (ESCA) (*p* = 0.001) and stomach adenocarcinoma (STAD) (*p* = 0.018); while GLS expression was down-regulated in breast invasive carcinoma (BRCA), kidney renal clear cell carcinoma (KIRC), kidney chromophobe (KICH), lung squamous cell carcinoma (LUSC) (*p* < 0.001) and prostate adenocarcinoma (PRAD) (*p* = 0.033) (Fig. 2C). Next, the corresponding normal tissues from the GTEx database were considered as controls for further evaluation. The evaluation outcomes indicated that GLS was up-regulated in COAD, CHOL, diffuse large B cell lymphoma (DLBC), ESCA, HNSC, acute myeloid leukemia (AML), LIHC, pancreatic adenocarcinoma (PAAD), rectal adenocarcinoma (READ), STAD and thymoma (THYM) (all *p* < 0.001); while GLS was down-regulated in adrenocortical carcinoma (ACC), BRCA, cervical squamous cell carcinoma and endocervical adenocarcinoma (CESC), glioblastoma multiforme (GBM), KICH, KIRC, brain low-grade glioma (LGG), lung adenocarcinoma (LUAD), LUSC, ovarian serous cystadenocarcinoma (OV), PRAD, skin cutaneous melanoma (SKCM), thyroid carcinoma (THCA), UCEC, uterine carcinosarcoma (UCS) (all *p* < 0.001), bladder urothelial carcinoma (BLCA) (*p* = 0.009) and pheochromocytoma and paraganglioma (PCPG) (*p* = 0.02) (Fig. 2D).

**Fig. 3:**
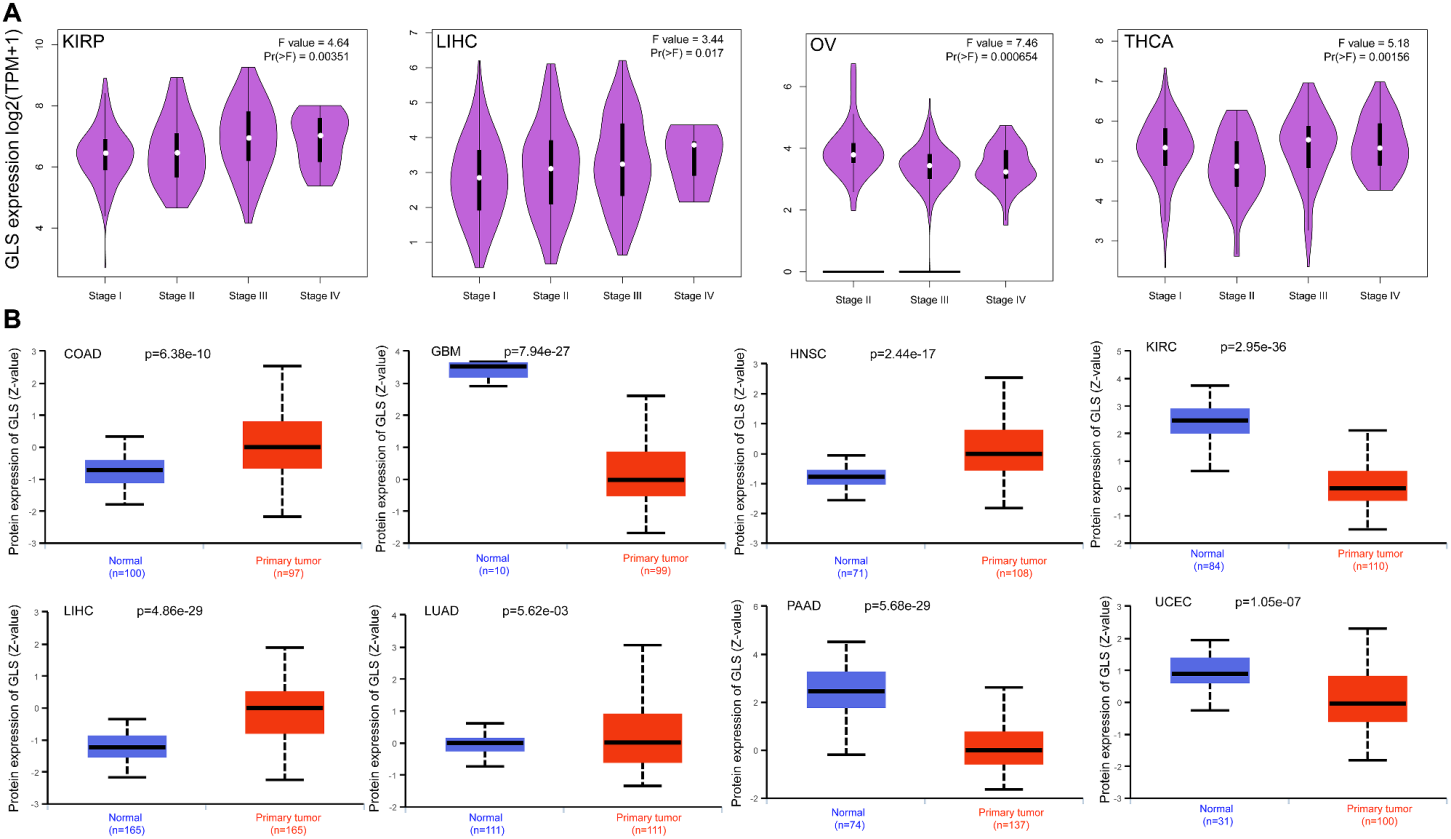
Differential expression of glutaminase (GLS) in pathology and proteomics (**A**) GLS expression in various pathological stages of pan-cancer; (**B**) Differential protein expression of GLS between tumor and normal tissues across pan-cancer. GLS, glutaminase. *P < 0.05, **P < 0.01, ***P < 0.001

The relationship between GLS expression and different pathological stages in pan-cancer was also investigated. As shown in Fig. 3A, significant differences were disclosed in KIRP, LIHC, OV and THCA (all *p* < 0.05) but not in others. As for GLS protein expression level, it was higher in the primary tumor tissues of COAD, HNSC, LIHC and LUAD (all *p* < 0.01) than in normal tissues, while opposites were observed in the GBM, KIRC, PAAD and UCEC (all *p* < 0.001) (Fig. 3B).

### Association between glutaminase (GLS) and molecular/immune subtypes in pan-cancer

**Fig. 4:**
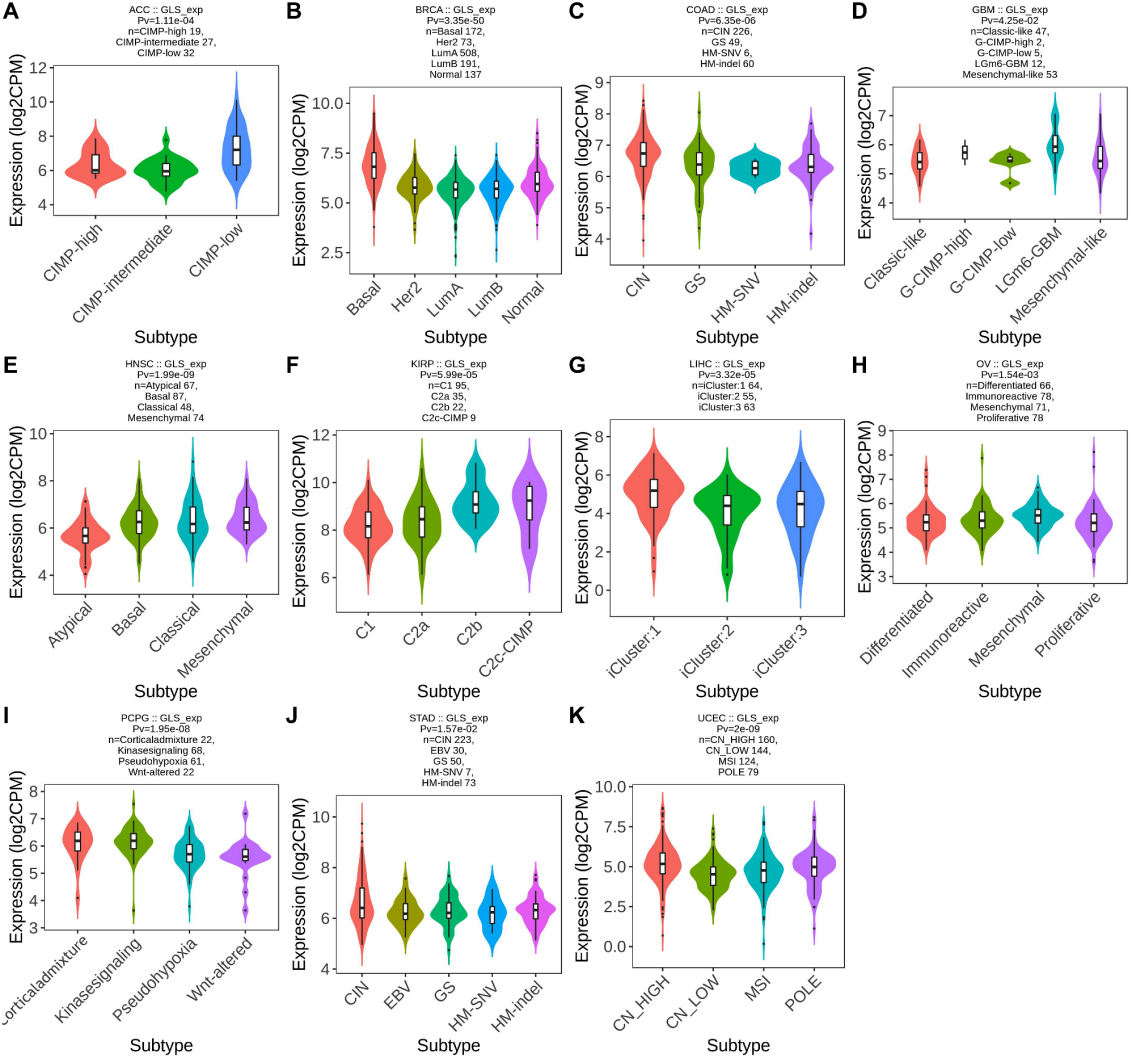
Correlations between glutaminase (GLS) expression and molecular subtypes across the cancer genome atlas (TCGA) cancers. (**A**) ACC; (**B**) BRCA; (**C**) COAD; (**D**) GBM; (**E**) HNSC; (**F**) KIRP; (**G**) LIHC; (**H**) OV; (**I**) PCPG; (**J**) STAD; (**K**) UCEC. *P < 0.05, **P < 0.01, ***P < 0.001

According to the TISIB database analysis, differential GLS expression existed in different molecular subtypes of eleven carcinoma types, including ACC, BRCA, COAD, GBM, HNSC, KIRP, LIHC, OV, PCPG, STAD and UCEC. Moreover, for ACC, GLS was expressed the highest in the molecular subtype of CIMP-low (*p* = 1.11e-04) (Fig. 4A). Besides, the highest GLS expression was observed in the molecular subtype of Basal for BRCA (*p* = 3.35e-50) (Fig. 4B) and the molecular subtype of CIN for COAD (*p* = 6.35e-06) (Fig. 4C). Concerning GBM, GLS was expressed the highest in the molecular subtype of LGm6-GBM (*p* = 4.25e-02) (Fig. 4D). As for HNSC, GLS was expressed less in the molecular subtype of Atypical than others (*p* = 1.99e-09) (Fig. 4E). The highest GLS expression was observed in the molecular subtype of C2c-CIMP for KIRP (*p* = 5.99e-05) (Fig. 4F) and the molecular subtype of iCluster:1 for LIHC (*p* = 3.32e-05) (Fig. 4G). For OV, the molecular subtype with the highest GLS expression was Mesenchymal (*p* = 1.54e-03) (Fig. 4H). For PCPG, GLS was expressed higher in the molecular subtypes of Corticaladmixture and Kinasesignaling than the molecular subtypes of Pseudohypoxia and Wnt-altered (*p* = 1.95e-08) (Fig. 4I). The highest expression of GLS was observed in the molecular subtype of CIN for STAD (*p* = 1.57e-02) (Fig. 4J). In contrast, the lowest GLS expression was found in the molecular subtype of CN_LOW for UCEC (*p* = 2e-09) (Fig. 4K).

**Fig. 5:**
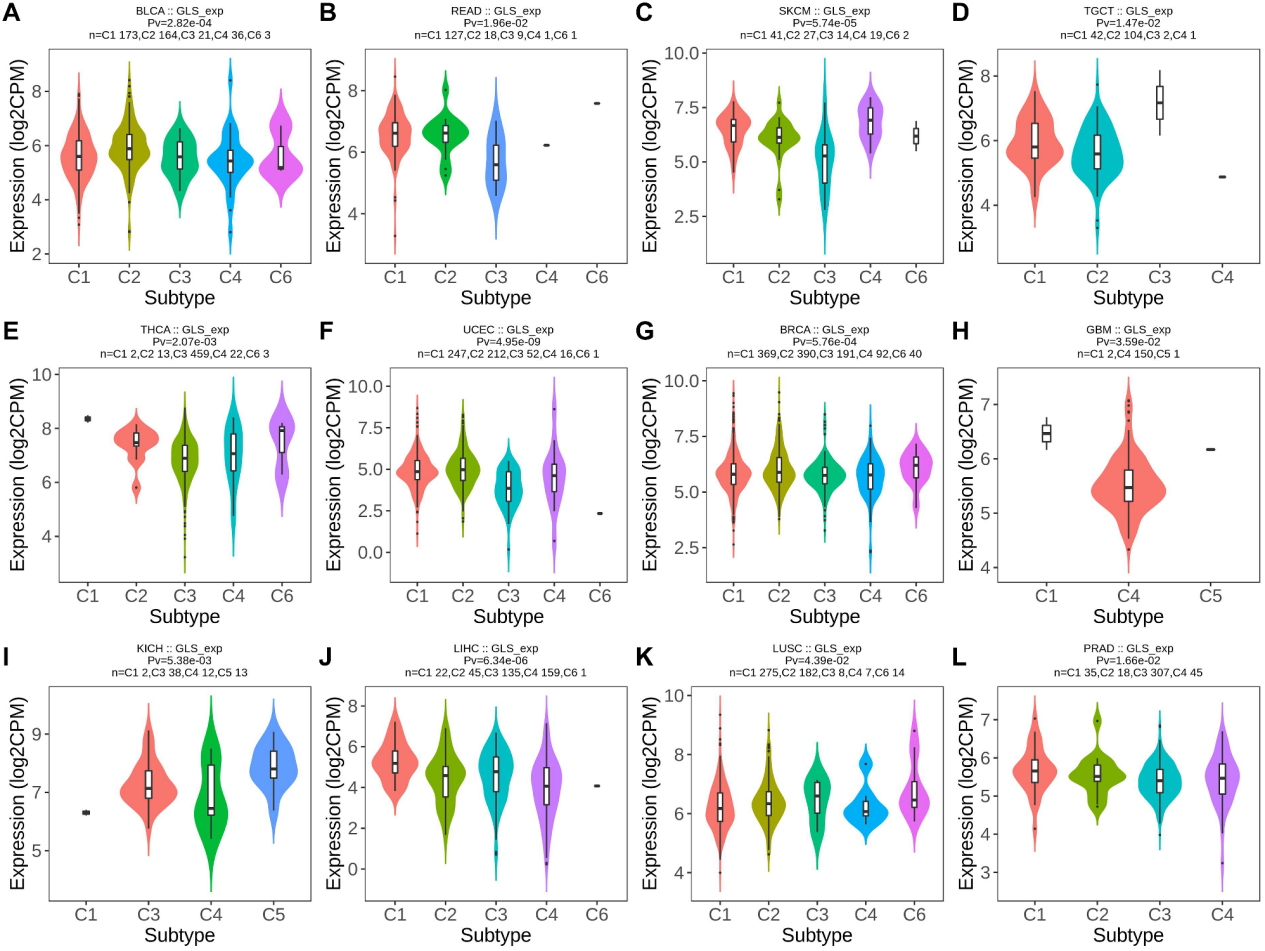
Correlations between glutaminase (GLS) expression and immune subtypes across the cancer genome atlas (TCGA) cancers. (**A**) BLCA; (**B**) READ; (**C**) SKCM; (**D**) TGCT; (**E**) THCA; (**F**) UCEC; (**G**) BRCA; (**H**) GBM; (**I**) KICH; (**J**) LIHC; (**K**) LUSC; (**L**) PRAD. *P < 0.05, **P < 0.01, ***P < 0.001

Furthermore, significant correlations between GLS expression and immune subtypes in twelve carcinoma types were exhibited. Briefly, GLS expression was highest in C2 (IFN-gamma dominant) for BLCA (*p* = 2.82e-04) (Fig. 5A), lowest in C3 (inflammatory) for READ (*p* = 1.96e-02) (Fig. 5B), highest in C4 (lymphocyte depleted) (*p* = 5.74e-05) (Fig. 5C), lowest in C2 for TGCT (*p* = 1.47e-02) (Fig. 5D), highest in C6 (TGF-b dominant) for THCA (*p* = 2.07e-03) (Fig. 5E), lowest in C3 for UCEC (*p* = 4.95e-09) (Fig. 5F), highest in C6 for BRCA (*p* = 5.76e-04) (Fig. 5G), lowest in C4 for GBM (*p* = 3.59e-02) (Fig. 5H), highest in C5 (immunologically quiet) for KICH (*p* = 5.38e-03) (Fig. 5I), highest in C1 (wound healing) for LIHC (*p* = 6.34e-06) (Fig. 5J), lowest in C4 for LUSC (*p* = 4.39e-02) (Fig. 5K) and highest in C1 for PRAD (*p* = 1.66e-02) (Fig. 5L).

### Diagnostic value of glutaminase (GLS) in pan-cancer

**Fig. 6:**
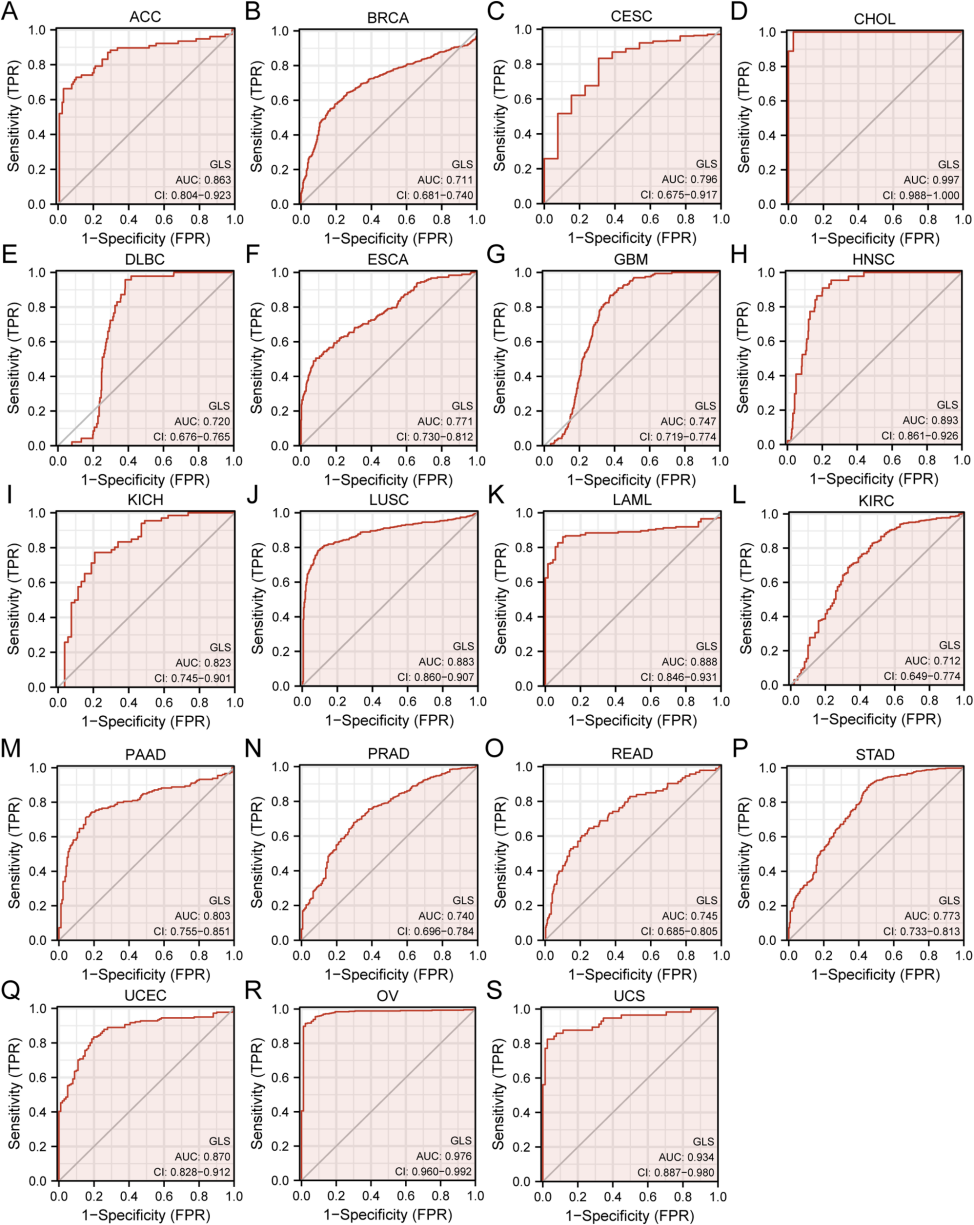
Diagnostic value of glutaminase (GLS) in pan-cancer. (**A**) ACC; (**B**) BRCA; (**C**) CESC; (**D**) CHOL; (**E**) DLBC; (**F**) ESCA; (**G**) GBM; (**H**) HNSC; (**I**) KICH; (**J**) LUSC; (**K**) LAML; (**L**) KIRC; (**M**) PAAD; (**N**) PRAD; (**O**) READ; (**P**) STAD; (**Q**) UCEC; (**R**) OV; (**S**) UCS. *P < 0.05, **P < 0.01, ***P < 0.001

The ROC curves were performed to evaluate the diagnostic capacity of GLS in pan-cancer. GLS was proved to be a reliable diagnostic biomarker in 19 carcinomas with a sure accuracy (AUC > 0.7), including ACC (AUC = 0.863, 95% CI = 0.804–0.923) (Fig. 6A), BRCA (AUC = 0.711, 95% CI = 0.681–0.740) (Fig. 6B), CESC (AUC = 0.796, 95% CI = 0.675–0.917) (Fig. 6C), CHOL (AUC = 0.997, 95% CI = 0.988–1.000) (Fig. 6D), DLBC (AUC = 0.720, 95% CI = 0.745–0.901) (Fig. 6E), ESCA (AUC = 0.771, 95% CI = 0.730–0.812) (Fig. 6F), GBM (AUC = 0.747, 95% CI = 0.719–0.774) (Fig. 6G), HNSC (AUC = 0.893, 95% CI = 0.861–0.926) (Fig. 6H), KICH (AUC = 0.823, 95% CI = 0.745–0.901) (Fig. 6I), LUSC (AUC = 0.883, 95% CI = 0.860–0.907) (Fig. 6J), AML (AUC = 0.888, 95% CI = 0.846–0.931) (Fig. 6K), KIRC (AUC = 0.712, 95% CI = 0.649–0.774) (Fig. 6L), PAAD (AUC = 0.803, 95% CI = 0.755–0.851) (Fig. 6M), PRAD (AUC = 0.740, 95% CI = 0.696–0.784) (Fig. 6N), READ (AUC = 0.745, 95% CI = 0.685–0.805) (Fig. 6O), STAD (AUC = 0.773, 95% CI = 0.733–0.813) (Fig. 6P), UCEC (AUC = 0.870, 95% CI = 0.828–0.912) (Fig. 6Q), OV (AUC = 0.976, 95% CI = 0.960–0.992) (Fig. 6R) and UCS (AUC = 0.934, 95% CI = 0.887–0.980) (Fig. 6S). Among them, a remarkable accuracy in diagnosis was observed predominantly in CHOL, OV and UCS (AUC > 0.9).

### Prognostic value of glutaminase (GLS) in pan-cancer

**Fig. 7:**
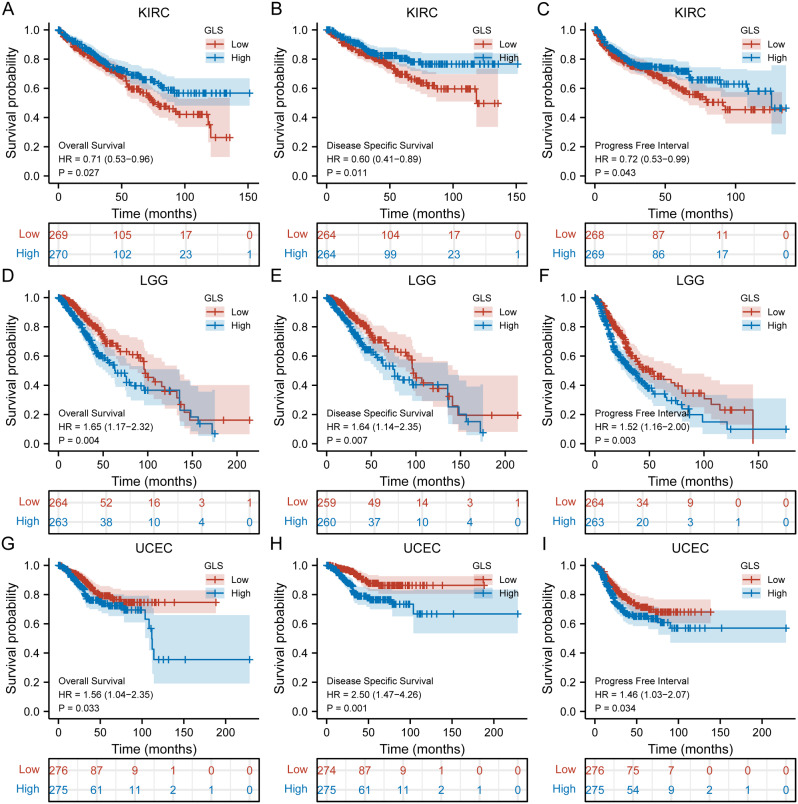
Prognostic value of glutaminase (GLS) in pan-cancer. (**A**–**C**) Prognostic value of GLS in KIRC (OS, DSS and PFI); (**D**–**F**) Prognostic value of GLS in LGG (OS, DSS and PFI); (**G**–**I**) Prognostic value of GLS in UCEC (OS, DSS and PFI). *P < 0.05, **P < 0.01, ***P < 0.001

After a thorough survival analysis of GLS in pan-cancer, we found that GLS expression level was associated prominently with the OS, DSS and PFI of KIRC, LGG and UCEC. With regard to KIRC, patients with higher expression of GLS had a better prognosis, including OS (HR = 0.71, 95% CI = 0.53–0.96, *p* = 0.027) (Fig. 7A), DSS (HR = 0.60, 95% CI = 0.41–0.89, *p* = 0.011) (Fig. 7B) and PFI (HR = 0.72, 95% CI = 0.53–0.99, *p* = 0.043) (Fig. 7C). However, about LGG, patients with higher expression of GLS had a worse prognosis, including OS (HR = 1.65, 95% CI = 1.17–2.32, *p* = 0.004) (Fig. 7D), DSS (HR = 1.64, 95% CI = 1.14–2.35, *p* = 0.007) (Fig. 7E) and PFI (HR = 1.52, 95% CI = 1.16–2.00, *p* = 0.003) (Fig. 7F). Also, for UCEC, patients with higher expression of GLS had a worse prognosis, including OS (HR = 1.56, 95% CI = 1.04–2.35, *p* = 0.033) (Fig. 7G), DSS (HR = 2.50, 95% CI = 1.47–4.26, *p* = 0.001) (Fig. 7H) and PFI (HR = 1.46, 95% CI = 1.03–2.07, *p* = 0.034) (Fig. 7I).

### Glutaminase (GLS)-ralated DNA methlytion analysis in uterine corpus endometrial carcinoma (UCEC) and kidney renal clear cell carcinoma (KIRC)

Following above comprehensive analyses in pan-cancer, we focused the DNA methylation analysis on UCEC and KIRC. Heatmaps were utilized to display the methylation status of different CpG sites in GLS in UCEC (Figure S1A) and KIRC (Figure S2A), along with clustering analysis of relevant clinical indicators. Further analysis revealed that increased methylation levels at the cg03962451 (HR = 1.975, *p* = 0.0063), cg04304216 (HR = 2.555, *p* = 0.00079), and cg17537719 (HR = 2.539, *p* = 0.0024) loci in UCEC were linked to a poor prognosis (Figure.S1B), whereas decreased methylation levels at the cg06552369 (HR = 0.446, *p* = 0.002), cg26332715 (HR = 0.391, *p* = 1.4e-05), and cg16975027 (HR = 0.544, *p* = 0.003) loci in KIRC were associated with poor prognosis (Figure.S2B).

### Glutaminase (GLS)-related protein-protein interaction (PPI) network construction and gene enrichment analysis

**Fig. 8:**
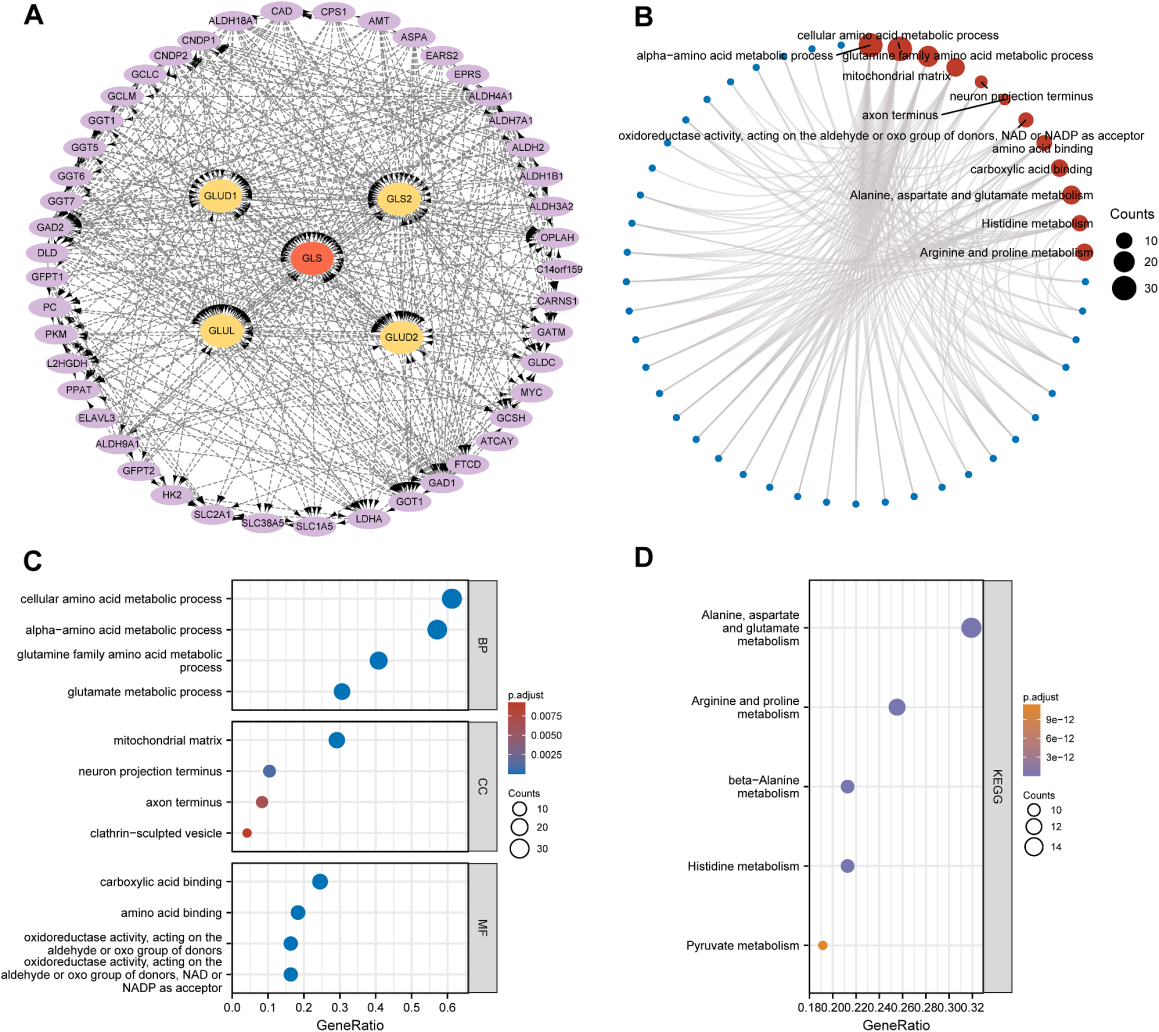
Protein-protein interaction (PPI) network and enrichment analysis of glutaminase (GLS). (**A**) PPI network of GLS; (**B**) Circle plot of GO and KEGG analyses; (**C**) GO analysis of top 4 pathways; (**D**) KEGG analysis of top 5 pathways. GO, Gene Ontology; KEGG, Kyoto Encyclopedia of Genes and Genomes. PPI, protein-protein interaction; GLS, glutaminase. *P < 0.05, **P < 0.01, ***P < 0.001

Based on the evidence of text mining, experiments and databases, the GLS-related PPI network of 50 predicted functional partners was constructed, of which the top 4 were highlighted (Fig. 8A). Next, the 50 GLS-related targeted proteins were subject to GO and KEGG enrichment analysis (Fig. 8B). As shown in Fig. 8C, the BP was mainly enriched in cellular amino acid metabolic process, alpha-amino acid metabolic process, glutamine family amino acid metabolic process and glutamate metabolic process. The CC was primarily involved in the mitochondrial matrix, neuron projection terminus, axon terminus and clathrin-sculpted vesicle. The chief MF was enriched in carboxylic acid binding, amino acid binding, and oxidoreductase activity, acting on the aldehyde or oxo group of donors, *NAD* or *NADP* as acceptor. The primary KEGG pathways contained alanine, aspartate and glutamate metabolism, arginine and proline metabolism, beta-alanine metabolism, histidine metabolism and pyruvate metabolism (Fig. 8D).

### Correlation between glutaminase (GLS) expression and various clinical characteristics in uterine corpus endometrial carcinoma (UCEC)

**Fig. 9:**
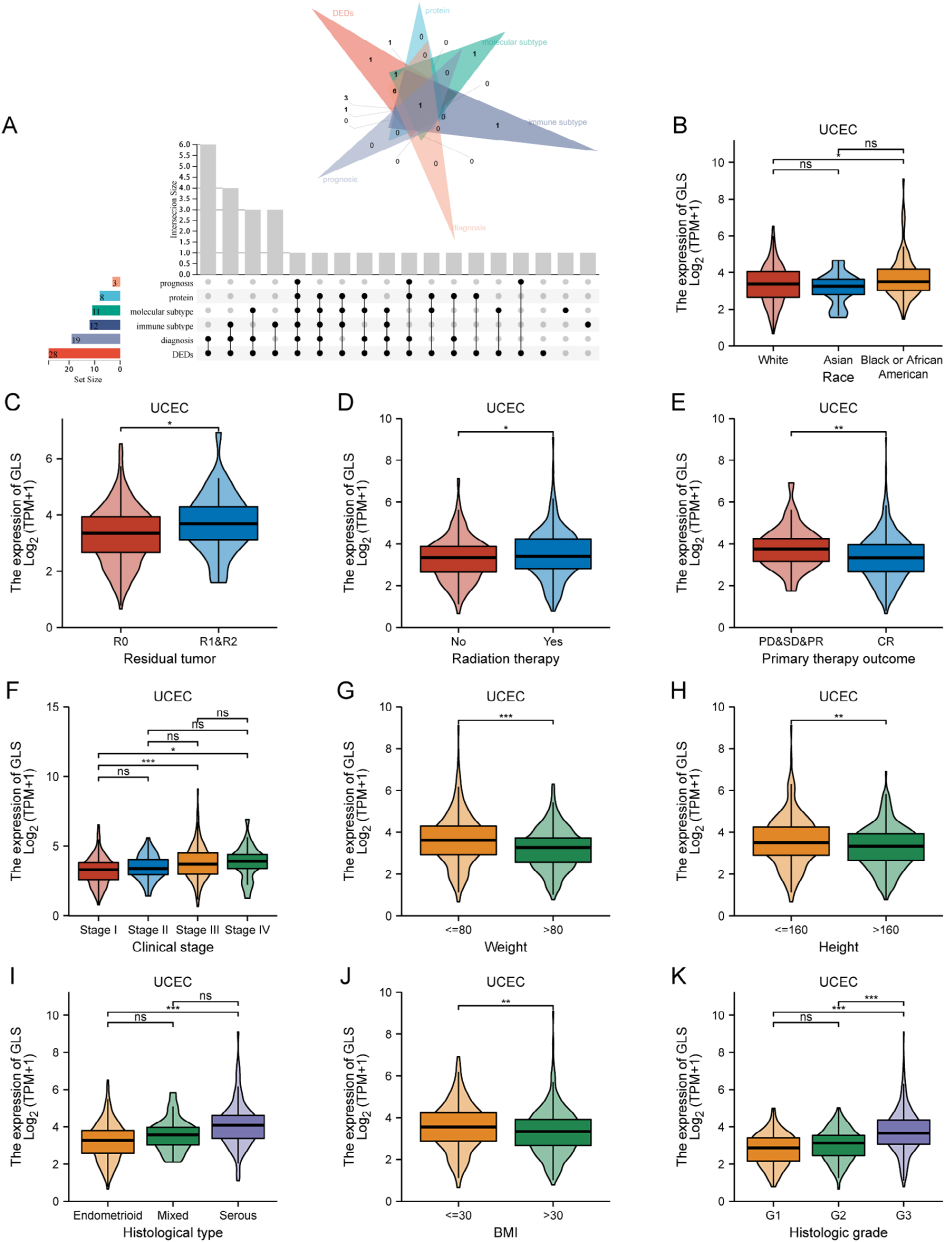
Clinical correlation analysis of glutaminase (GLS) expression in uterine corpus endometrial carcinoma (UCEC). (**A**) Upset diagram for identifying cancers associated with GLS expression; (**B**) Clinical association between GLS and race; (**C**) Clinical association between GLS and residual tumor; (**D**) Clinical association between GLS and radiation therapy; (**E**) Clinical association between GLS and primary therapy outcome; (**F**) Clinical association between GLS and clinical stage; (**G**) Clinical association between GLS and weight; (**H**) Clinical association between GLS and height. (**I**) Clinical association between GLS and histological type; (**J**) Clinical association between GLS and BMI; (**K**) Clinical association between GLS and histologic grade. GLS, glutaminase. *P < 0.05, **P < 0.01, ***P < 0.001

According to the comprehensive analysis above, we discovered that GLS expression was notably associated with UCEC (Fig. 9A). Therefore, we further explored the correlation between GLS and different clinical features in UCEC. Based on the baseline characteristics of patients summarized in Table 1, there was a significant difference in GLS expression level with respect to clinical stage, weight, histological type, histologic grade (all *p* < 0.001), primary therapy outcome (*p* = 0.001), height (*p* = 0.013) and body mass index (BMI) (*p* = 0.003). Moreover, GLS expression was higher in patients of black or African Americans than whites (*p* < 0.05) (Fig. 9B). Patients with R0 resection (*p* < 0.05) (Fig. 9C), no radiation therapy (*p* < 0.05) (Fig. 9D) and primary therapy outcome (complete response, CR) (*p* < 0.01) (Fig. 9E) were related to lower GLS expression, while patients with clinical stage III/IV (*p* < 0.05) (Fig. 9F), weight > 80 (*p* < 0.001) (Fig. 9G), height ≤ 160 (*p* < 0.01) (Fig. 9H), histological type of serous (*p* < 0.001) (Fig. 9I), BMI ≤ 30 (*p* < 0.01) (Fig. 9J) and histological grade 3 (*p* < 0.001) (Fig. 9K) were related to higher GLS expression, respectively.

**Table 1:** Baseline characteristics of patients (TCGA-UCEC).

Characteristics	Levels	Low expression of GLS	High expression of GLS	*p* value*
		*N*=276	*N*=276	
Clinical stage, n (%)	Stage I	194 (70.3%)	148 (53.6%)	**< 0.001**
	Stage II	27 (9.8%)	24 (8.7%)	
	Stage III	47 (17%)	83 (30.1%)	
	Stage IV	8 (2.9%)	21 (7.6%)	
Primary therapy outcome, n (%)	PD	10 (4%)	10 (4.3%)	**0.001**
	SD	3 (1.2%)	3 (1.3%)	
	PR	0 (0%)	12 (5.2%)	
	CR	236 (94.8%)	206 (89.2%)	
Weight, n (%)	<=80	97 (36.6%)	146 (55.5%)	**< 0.001**
	>80	168 (63.4%)	117 (44.5%)	
Height, n (%)	<=160	109 (41.6%)	138 (52.9%)	**0.013**
	>160	153 (58.4%)	123 (47.1%)	
BMI, n (%)	<=30	89 (34.2%)	123 (47.5%)	**0.003**
	>30	171 (65.8%)	136 (52.5%)	
Histological type, n (%)	Endometrioid	237 (85.9%)	173 (62.7%)	**< 0.001**
	Mixed	9 (3.3%)	15 (5.4%)	
	Serous	30 (10.9%)	88 (31.9%)	
Histologic grade, n (%)	G1	73 (26.8%)	25 (9.3%)	**< 0.001**
	G2	83 (30.5%)	37 (13.8%)	
	G3	116 (42.6%)	207 (77%)	
Age, n (%)	<=60	109 (39.6%)	97 (35.4%)	0.349
	>60	166 (60.4%)	177 (64.6%)	
Residual tumor, n (%)	R0	199 (93.4%)	176 (88%)	0.075
	R1	10 (4.7%)	12 (6%)	
	R2	4 (1.9%)	12 (6%)	
Tumor invasion, n (%)	<50	143 (56.7%)	116 (52.3%)	0.374
	>=50	109 (43.3%)	106 (47.7%)	
Menopause status, n (%)	Pre	16 (6.3%)	19 (7.5%)	0.186
	Peri	5 (2%)	12 (4.7%)	
	Post	232 (91.7%)	222 (87.7%)	
Hormones therapy, n (%)	No	158 (88.3%)	139 (84.2%)	0.353
	Yes	21 (11.7%)	26 (15.8%)	
Diabetes, n (%)	No	165 (72.1%)	163 (73.4%)	0.825
	Yes	64 (27.9%)	59 (26.6%)	
Radiation therapy, n (%)	No	147 (54.9%)	132 (51%)	0.420
	Yes	121 (45.1%)	127 (49%)	
Surgical approach, n (%)	Minimally Invasive	98 (36.4%)	110 (42.1%)	0.208
	open	171 (63.6%)	151 (57.9%)	
Race, n (%)	Asian	13 (5.1%)	7 (2.8%)	0.100
	Black or African American	46 (18%)	62 (24.6%)	
	White	196 (76.9%)	183 (72.6%)	

Data are presented as n (%). UCEC, uterine corpus endometrial carcinoma. *Compared with each group (Fisher exact test, or Pearson’s chi-square test). *p* < 0.05 was considered statistically significant (highlighted in bold)

### Correlation between glutaminase (GLS) expression and subgroup prognosis in UCEC

**Fig. 10:**
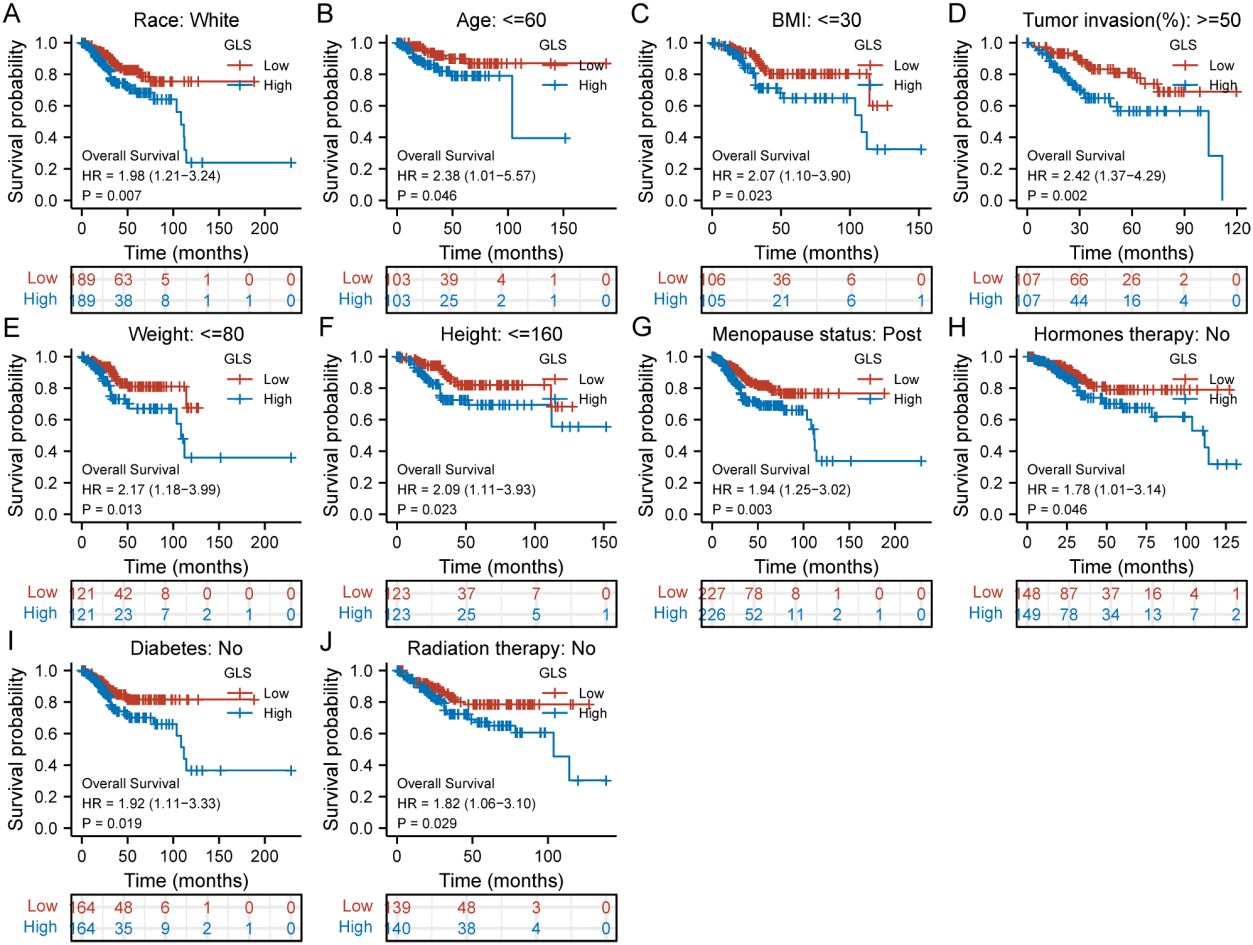
Subgroup analyses on overall survival (OS) of glutaminase (GLS) expression in TCGA-UCEC. (**A**) Race (White); (**B**) Age ≤ 60; (**C**) BMI ≤ 30; (**D**) Tumor invasion ≥ 50%; (**E**) Weight ≤ 80; (**F**) Height ≤ 160; (**G**) Menopause status (Post); (**H**) Hormones therapy (No); (**I**) Diabetes (No); (**J**) Radiation therapy (No). BMI, body mass index. *P < 0.05, **P < 0.01, ***P < 0.001

We assessed the associations between GLS and prognosis (OS, DSS and PFI) of different clinical subgroups in UCEC. The K-M plots revealed that higher GLS expression was related to a worse OS in a subgroup of race of white (HR = 1.98, *p* = 0.007) (Fig. 10A), a subgroup of age ≤ 60 (HR = 2.38, *p* = 0.046) (Fig. 10B), a subgroup of BMI ≤ 30 (HR = 2.07, *p* = 0.023) (Fig. 10C), a subgroup of tumor invasion ≥ 50% (HR = 2.42, *p* = 0.002) (Fig. 10D), a subgroup of weight ≤ 80 (HR = 2.17, *p* = 0.013) (Fig. 10E), a subgroup of height ≤ 160 (HR = 2.09, *p* = 0.023) (Fig. 10F), a subgroup of post menopause (HR = 1.94, *p* = 0.003) (Fig. 10G), a subgroup of hormones-free therapy (HR = 1.78, *p* = 0.046) (Fig. 10H), a subgroup of diabetes-free (HR = 1.92, *p* = 0.019) (Fig. 10I) and a subgroup of non-radiation therapy (HR = 1.82, *p* = 0.029) (Fig. 10J).

**Fig. 11:**
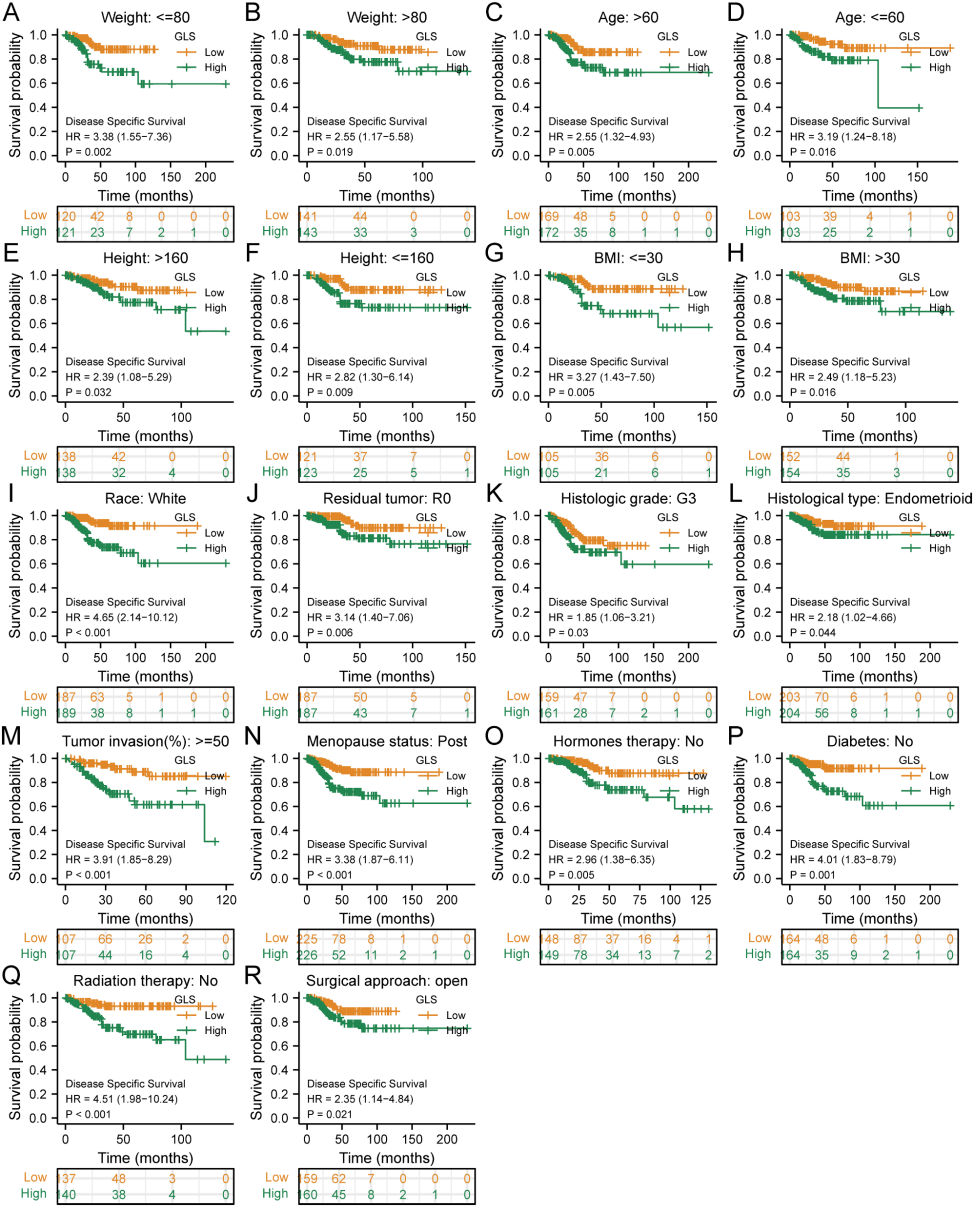
Subgroup analyses on disease-specific survival (DSS) of glutaminase (GLS) expression in TCGA-UCEC. (**A**) Weight ≤ 80; (**B**) Weight > 80; (**C**) Age > 60; (**D**) Age ≤ 60; (**E**) Height > 160; (**F**) Height ≤ 160; (**G**) BMI ≤ 30; (**H**) BMI > 30; (**I**) Race (White); (**J**) Residual tumor (R0); (**K**) Histologic grade (G3); (**L**) Histological type (Endometrioid); (**M**) Tumor invasion ≥ 50%; (**N**) Menopause status (Post); (**O**) Hormones therapy (No); (**P**) Diabetes (No); (**Q**) Radiation therapy (No); (**R**) Surgical approach (Open). BMI, body mass index. *P < 0.05, **P < 0.01, ***P < 0.001

With regard to DSS, elevated GLS expression was correlated with poorer prognosis of most clinical subgroups, including weigh ≤ 80 (HR = 3.38, *p* = 0.002) (Fig. 11A) or weight > 80 (HR = 2.55, *p* = 0.019) (Fig. 11B), age > 60 (HR = 2.55, *p* = 0.005) (Fig. 11C) or age ≤ 60 (HR = 3.19, *p* = 0.016) (Fig. 11D), height > 160 (HR = 2.39, *p* = 0.032) (Fig. 11E) or height ≤ 160 (HR = 2.82, *p* = 0.009) (Fig. 11F), BMI ≤ 30 (HR = 3.27, *p* = 0.005) (Fig. 11G) or BMI > 30 (HR = 2.49, *p* = 0.016) (Fig. 11H), race of white (HR = 4.65, *p* < 0.001) (Fig. 11I), R0 resection (HR = 3.14, *p* = 0.006) (Fig. 11J), histologic grade G3 (HR = 1.85, *p* = 0.03) (Fig. 11K), histological type of endometrioid (HR = 2.18, *p* = 0.044) (Fig. 11L), tumor invasion ≥ 50% (HR = 3.91, *p* < 0.001) (Fig. 11M), post menopause (HR = 3.38, *p* < 0.001) (Fig. 11N), hormones-free therapy (HR = 2.96, *p* = 0.005) (Fig. 11O), diabetes-free (HR = 4.01, *p* = 0.001) (Fig. 11P), non-radiation therapy (HR = 4.51, *p* < 0.001) (Fig. 11Q) and open surgical approach (HR = 2.35, *p* = 0.021) (Fig. 11R).

**Fig. 12:**
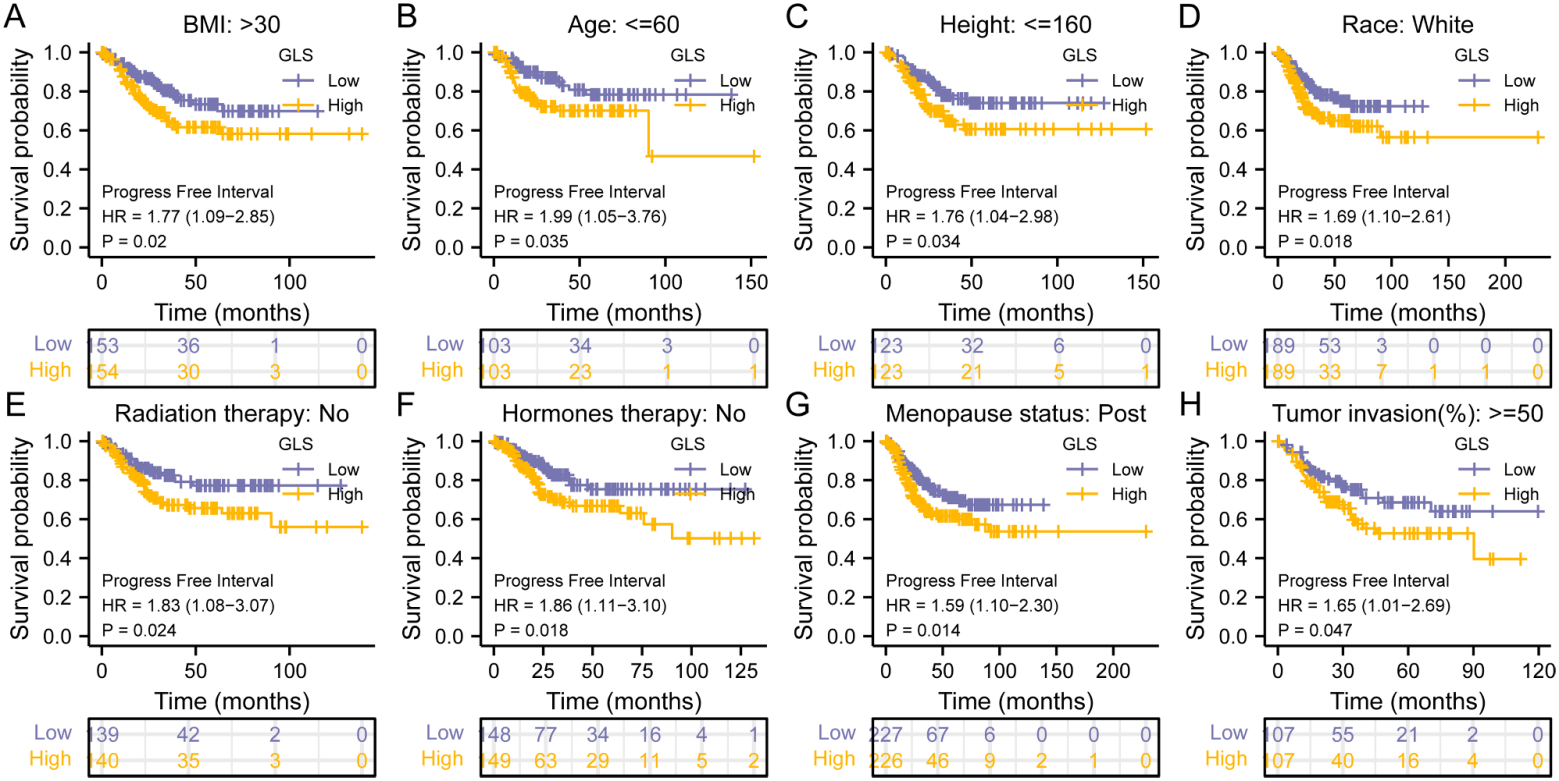
Subgroup analyses on progression-free interval of glutaminase (GLS) expression in TCGA-UCEC. (**A**) BMI > 30; (**B**) Age ≤ 60; (**C**) Height ≤ 160; (**D**) Race (White); (**E**) Radiation therapy (No); (**F**) Hormones therapy (No); (**G**) Menopause status (Post); (**H**) Tumor invasion ≥ 50%. BMI, body mass index. *P < 0.05, **P < 0.01, ***P < 0.001

For PFI, elevated GLS expression was also correlated with poorer prognosis of most clinical subgroups, including BMI > 30 (HR = 1.77, *p* = 0.02) (Fig. 12A), age ≤ 60 (HR = 1.99, *p* = 0.035) (Fig. 12B), height ≤ 160 (HR = 1.76, *p* = 0.034) (Fig. 12C), race of white (HR = 1.69, *p* = 0.018) (Fig. 12D), non-radiation therapy (HR = 1.83, *p* = 0.024) (Fig. 12E), hormones-free treatment (HR = 1.86, *p* = 0.018) (Fig. 12F), post menopause (HR = 1.59, *p* = 0.014) (Fig. 12G) and tumor invasion ≥ 50% (HR = 1.65, *p* = 0.047) (Fig. 12H).

### Establishment and assessment of glutaminase (GLS)-related nomogram

All clinical variables were included in the univariate analysis with respect to OS. The analysis results revealed that primary therapy outcome, R1&R2 resection, histologic grade G2&G3, tumor invasion ≥ 50% (all *p* < 0.001), non-radiation therapy (*p* = 0.018) and GLS expression (*p* = 0.002) were independent risk factors affecting the OS of patients with UCEC (Table 2). Furthermore, the outcomes of multivariate cox regression analysis disclosed that worse OS in UCEC was significantly correlated with primary therapy outcome (HR = 4.032, 95% CI = 1.550–10.490, *p* = 0.004), R1&R2 resection (HR = 2.75, 95% CI = 1.234–6.135, *p* = 0.013), histologic grade G2&G3 (HR = 11.982, 95% CI = 1.612–89.035, *p* = 0.015), non-radiation therapy (HR = 3.277, 95% CI = 1.743–6.163, *p* < 0.001) and GLS expression (HR = 1.384, 95% CI = 1.032–1.855, *p* = 0.030) (Table 2).

**Table 2:** The univariate and multivariate analysis for the OS (TCGA-UCEC).

Characteristics		Total(N)	Univariate analysis		Multivariate analysis	
			Hazard ratio (95% CI)	P value*	Hazard ratio (95% CI)	P value*
Primary therapy outcome	CR&PR&SD	460	Reference		NA	
	PD	20	7.821 (4.267-14.336)	**<0.001**	4.032 (1.550-10.490)	**0.004**
Residual tumor	R0	374	Reference		NA	
	R1&R2	38	3.101 (1.768-5.440)	**<0.001**	2.751 (1.234-6.135)	**0.013**
Histologic grade	G1	98	Reference		NA	
	G3&G2	442	11.604 (2.855-47.167)	**<0.001**	11.982 (1.612-89.035)	**0.015**
Radiation therapy	Yes	248	Reference		NA	
	No	279	1.684 (1.092-2.596)	**0.018**	3.277 (1.743-6.163)	**<0.001**
Tumor invasion(%)	<50	259	Reference		NA	
	>=50	214	2.813 (1.744-4.535)	**<0.001**	1.656 (0.838-3.274)	0.147
GLS		551	1.296 (1.097-1.532)	**0.002**	1.384 (1.032-1.855)	**0.030**

OS, overall survival; CI, confidence interval; NA, reference group or could not be evaluated. *Compared with each group (Log-Rank test or Omnibus test for univariate, Cox regression analysis with adjusted hazard for multivariate). *p*<0.05 means statistically significant (highlighted in bold)

**Fig. 13:**
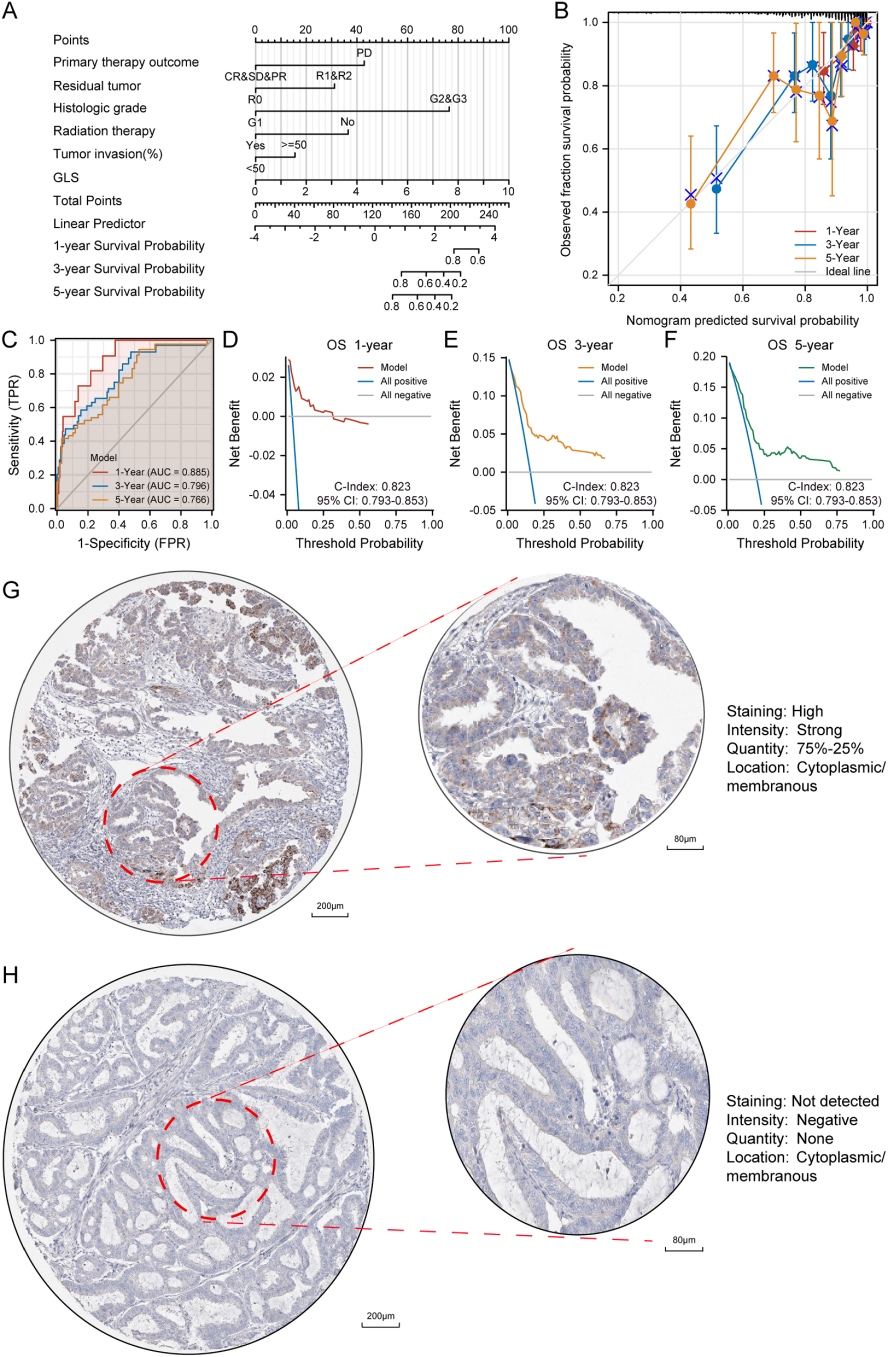
Establishment and assessment of glutaminase (GLS)-based nomogram for overall survival (OS) in TCGA-UCEC. (**A**) Glutaminase (GLS)-based nomogram included with 6 clinical components predicting 1,3, and 5-year OS; (**B**) Nomogram calibration curve for 1,3, and 5-year; (**C**) The time-dependent ROC curve of the prognostic model for predicting 1,3, and 5-year OS; (**D**–**F**) Decision curve analysis for evaluating the net benefits of nomogram at 1,3, and 5 years; (**G**) Immunohistochemical analysis of UCEC tissues with high GLS expression; (**H**) Immunohistochemical analysis of UCEC tissues with low GLS expression. OS, overall survival; ROC, receiver operating characteristic; UCEC, uterine corpus endometrial carcinoma. *P < 0.05, **P < 0.01, ***P < 0.001

Based on the results above, GLS and different clinical characteristics were integrated to construct a nomogram for predicting the prognosis of UCEC patients (Fig. 13A). The prognostic score could be calculated to predict the 1–5 years OS of UCEC patients, and the nomogram calibration curves (Fig. 13B) demonstrated an above average accuracy of the model. Further, time-dependent ROC curves were employed for further assessment of the accuracy of the model (1-year: AUC = 0.885, 3-year: AUC = 0.796, 5-year: AUC = 0.766) (Fig. 13C). In addition, the DCA showed an excellent clinical utility of the prognostic model (C-Index = 0.823, 95% CI = 0.793–0.853) (Fig. 13D–F).

As a supplement, we also compared the immunohistochemical results between UCEC tissues with high GLS expression (Fig. 13G) and those with low GLS expression (Fig. 13H) via the HPA database.

## Glutaminase (GLS)-related co-expression gene analysis in uterine corpus endometrial carcinoma (UCEC)

**Fig. 14:**
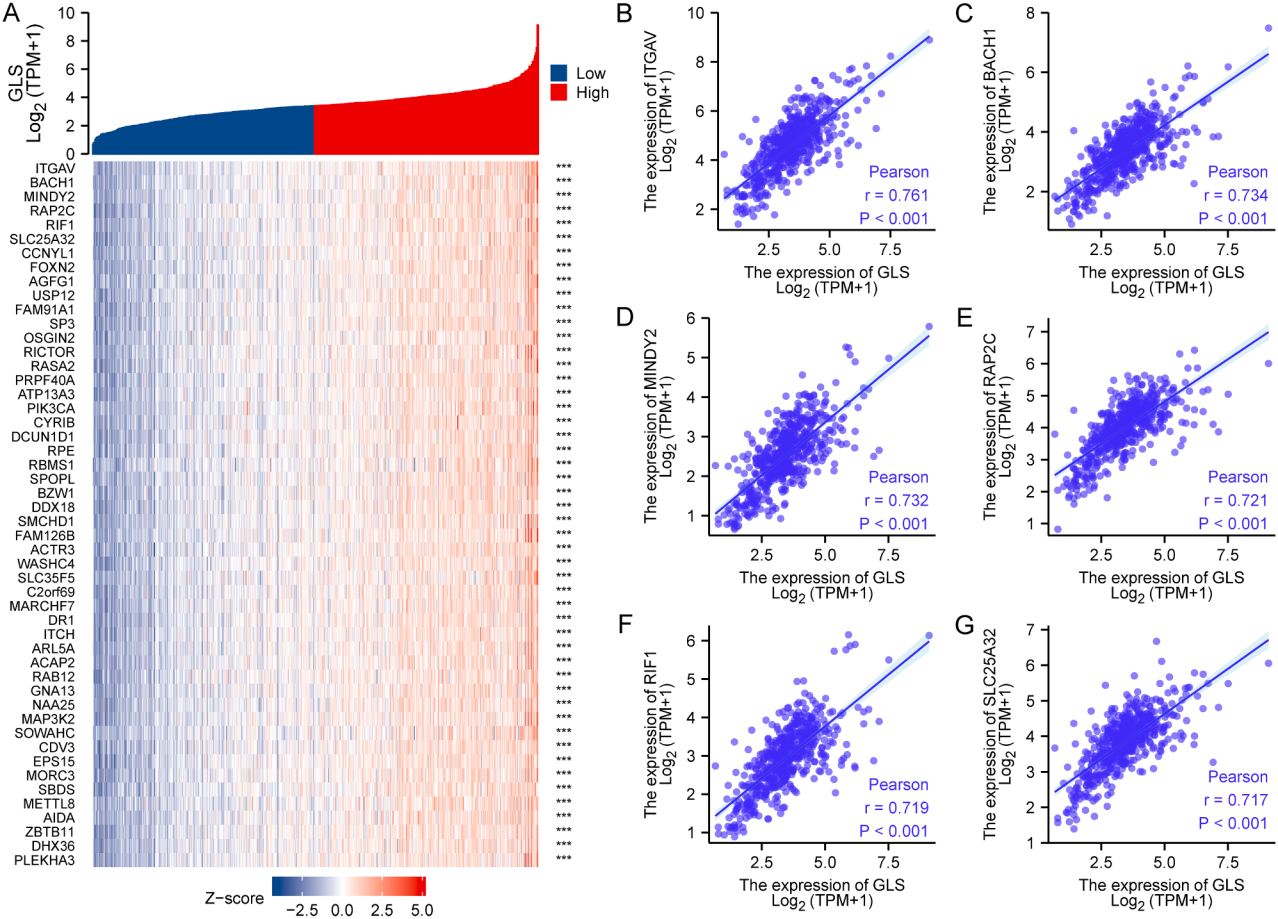
Top 50 genes positively correlated with glutaminase (GLS) expression in uterine corpus endometrial carcinoma (UCEC). (**A**) The heatmap of the top 50 co-expressed genes positively correlated with GLS in TCGA-UCEC; (**B**–**G**) Co-expressed analysis of the top 6 genes positively correlated with GLS in scatter plot. GLS, glutaminase. *P < 0.05, **P < 0.01, ***P < 0.001

**Fig. 15:**
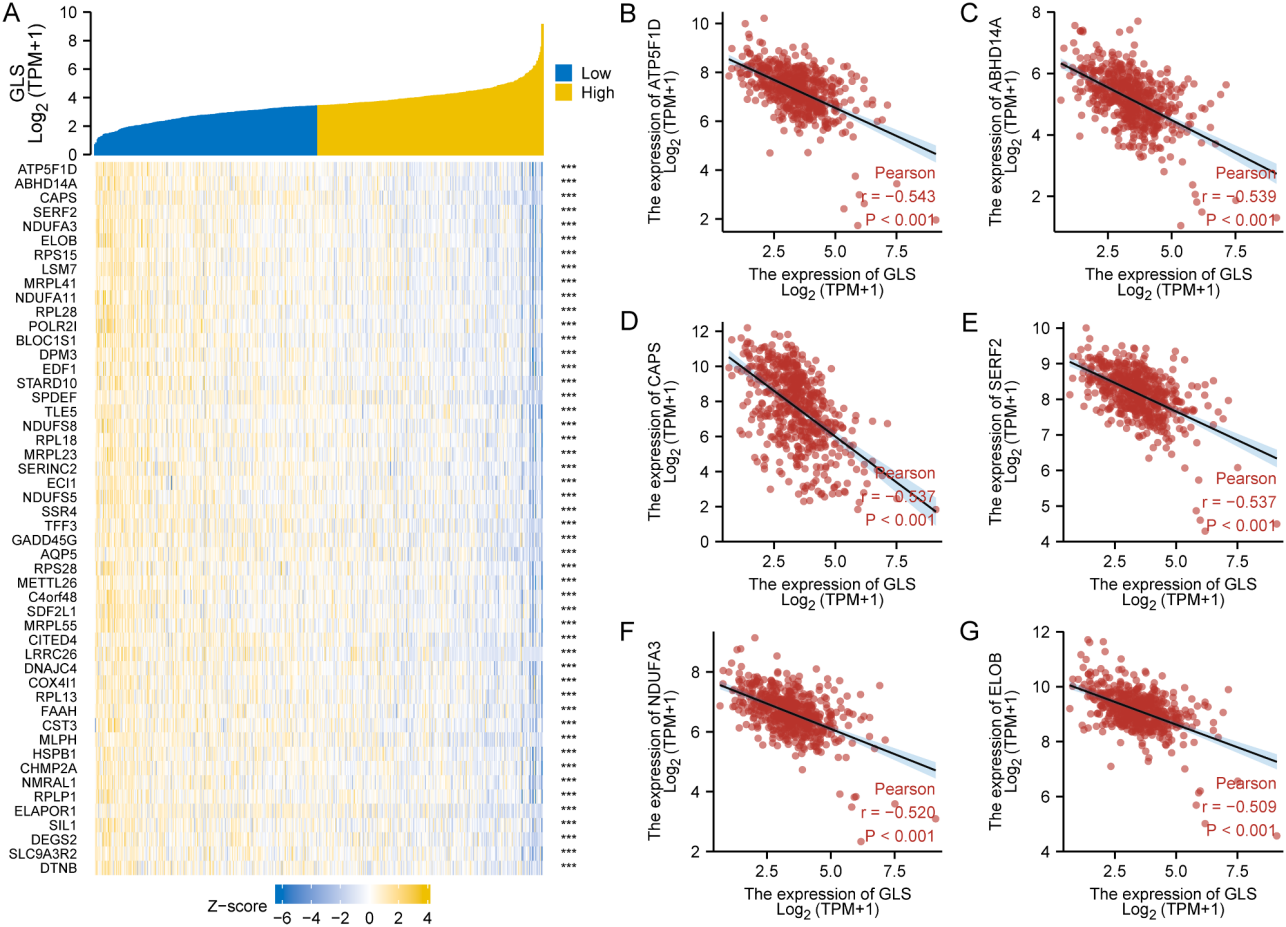
Top 50 genes negatively correlated with glutaminase (GLS) expression in uterine corpus endometrial carcinoma (UCEC). (**A**) The heatmap of the top 50 co-expressed genes negatively correlated with GLS in TCGA-UCEC; (**B**–**G**) Co-expressed analysis of the top 6 genes negatively correlated with GLS in scatter plot. GLS, glutaminase. *P < 0.05, **P < 0.01, ***P < 0.001

The top 50 genes positively/negatively associated with GLS expression in UCEC were acquired via co-expression gene analysis. These correlations were displayed in a heatmap (Fig. 14A) and the top 6 positively correlated co-expression genes were shown in scatter plots, including *ITGAV* (*r* = 0.761, *p* < 0.001) (Fig. 14B), *BACH1* (*r* = 0.734, *p* < 0.001) (Fig. 14C), *MINDY2* (*r* = 0.732, *p* < 0.001) (Fig. 14D), *RAP2C* (*r* = 0.721, *p* < 0.001) (Fig. 14E), *RIF1* (*r* = 0.719, *p* < 0.001) (Fig. 14F) and *SLC25A32* (*r* = 0.717, *p* < 0.001) (Fig. 14G). The negative correlations were summarized in Fig. 15A. The top 6 negatively correlated co-expression genes were displayed as follows: *ATP5F1D* (*r* = -0.543, *p* < 0.001) (Fig. 15B), *ABHD14A* (*r* = -0.539, *p* < 0.001) (Fig. 15C), *CAPS* (*r* = -0.537, *p* < 0.001) (Fig. 15D), *SERF2* (*r* = -0.537, *p* < 0.001) (Fig. 15E), *NDUFA3* (*r* = -0.520, *p* < 0.001) (Fig. 15F) and *ELOB* (*r* = -0.509, *p* < 0.001) (Fig. 15G).

### Glutaminase (GLS)-related DEGs and enrichment analysis in uterine corpus endometrial carcinoma (UCEC)

**Fig. 16:**
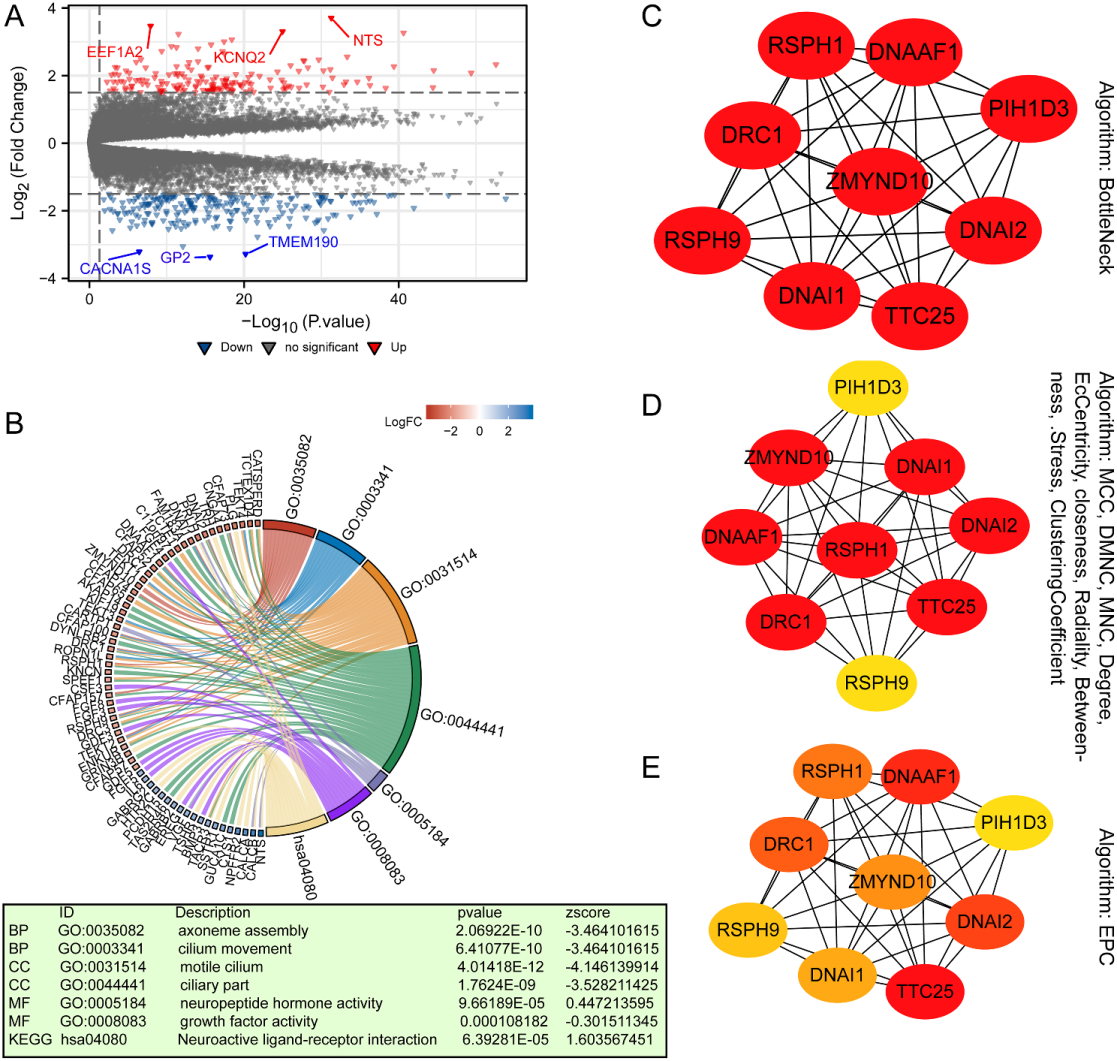
Establishment of PPI network and enrichment analyses on differentially expressed genes (DEGs) screened between glutaminase (GLS) high and low expression groups in uterine corpus endometrial carcinoma (UCEC). (**A**) The volcano plot of DEGs; (**B**) The GO and KEGG analyses of DEGs; (**C**–**E**) Hub genes and PPI network identified by using 12 algorithms. DEGs, differentially expressed genes; GO, Gene Ontology; KEGG, Kyoto Encyclopedia of Genes and Genomes; PPI, protein-protein interaction. *P < 0.05, **P < 0.01, ***P < 0.001

Next, the DEGs between GLS low-expression and high-expression groups in UCEC were explored. To be specific, a total of 398 DEGs were obtained, including 171 up-regulated genes and 227 down-regulated genes (Fig. 16A). These genes were subject to GO and KEGG analysis for exploring GLS-related particular pathways enriched in UCEC. As shown in Fig. 16B, the BP was primarily involved in axoneme assembly and cilium movement. The CC was mainly enriched in motile cilium and ciliary part. The primary MF contained neuropeptide hormone activity and growth factor activity, and the KEGG was mostly related to neuroactive ligand-receptor interaction.

Additionally, we used 12 algorithms to mine the potential hub genes from the DEGs. The top 9 hub genes, including *RSPH1*, *DNAAF1*, *PIH1D3*, *DRC1*, *ZMYND10*, DNAI2, *RSPH9*, *DNAI1* and *TTC25*, were obtained via the algorithm of bottleneck (Fig. 16C). Through the algorithms of MCC, DMNC, MNC, Degree, EcCentricity, closeness, Radiality, Between-ness, Stress, and Clustering Coefficient, we acquired the consistent result that the top 7 hub genes were *RSPH1*, *DNAAF1*, *DRC1*, *ZMYND10*, *DNAI2*, *DNAI1* and *TTC25* (Fig. 16D). Among them, the top 3 hub genes, namely, *TTC25*, *DNAAF1* and *DNAI2*, was calculated by the algorithm of EPC (Fig. 16E).

### Glutaminase (GLS)-related immune cells infiltration in pan-cancer

**Fig. 17:**
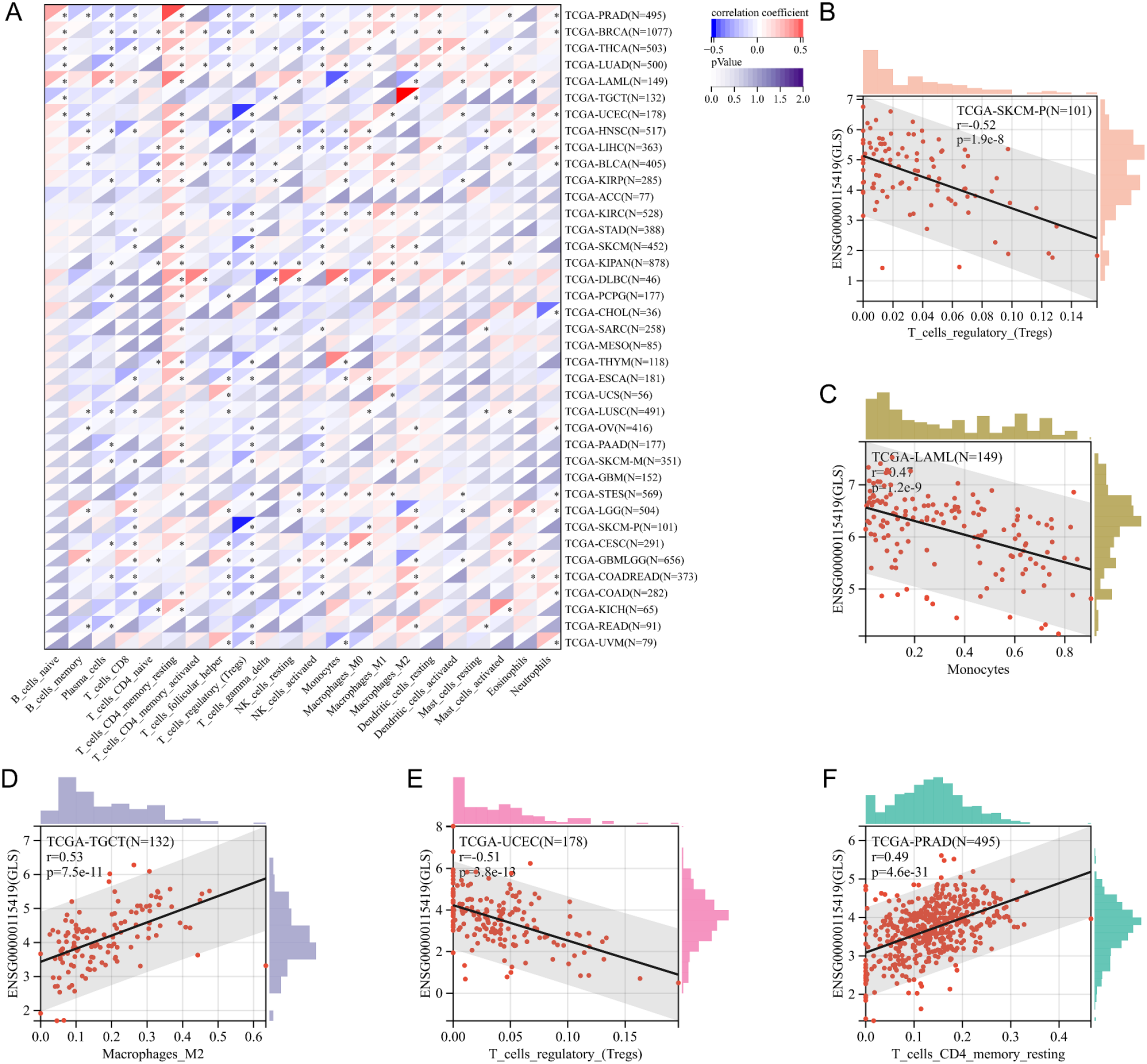
Correlation analysis of glutaminase (GLS) expression and Immune cells in pan-cancer. (**A**) The heatmap of GLS-immune cells correlation analysis; (**B**) Correlation analysis of GLS and Tregs in TCGA-SKCM; (**C**) Correlation analysis of GLS and monocytes in TCGA-AML; (**D**) Correlation analysis of GLS and Macrophages M2 in TCGA-TGCT; (**E**) Correlation analysis of GLS and Tregs in TCGA-UCEC; (**F**) Correlation analysis of GLS and CD4 + T cells in TCGA-PRAD; Tregs, T cells regulatory. GLS, glutaminase. *P < 0.05, **P < 0.01, ***P < 0.001

We obtained immune cells infiltration scores for 22 of 9554 tumor samples from 39 tumor types via the CIBERSORT algorithm. According to Pearson’s correlation analysis, we finally observed that GLS expression was significantly correlated with immune cells infiltration in 36 cancer species (Fig. 17A). Of them, the most significant correlations were displayed using scatter plots, including the correlations of GLS with T cells regulatory (Tregs) in TCGA-SKCM-primary (TCGA-SKCM-P) (*r* = -0.52, *p* = 1.9e-8) (Fig. 17B), monocytes in TCGA-AML (*r* = -0.47, *p* = 1.2e-9) (Fig. 17C), Marrophages_M2 in TCGA-TGCT (*r* = 0.53, *p* = 7.5e-11) (Fig. 17D), Tregs in TCGA-UCEC (*r* = -0.51, *p* = 3.8e-13) (Fig. 17E) and T_cells_CD4_memory_resting in TCGA-PRAD (*r* = 0.49, *p* = 4.6e-31) (Fig. 17F).

### Glutaminase (GLS)-related genomic heterogeneity and tumor stemness in pan-cancer

**Fig. 18:**
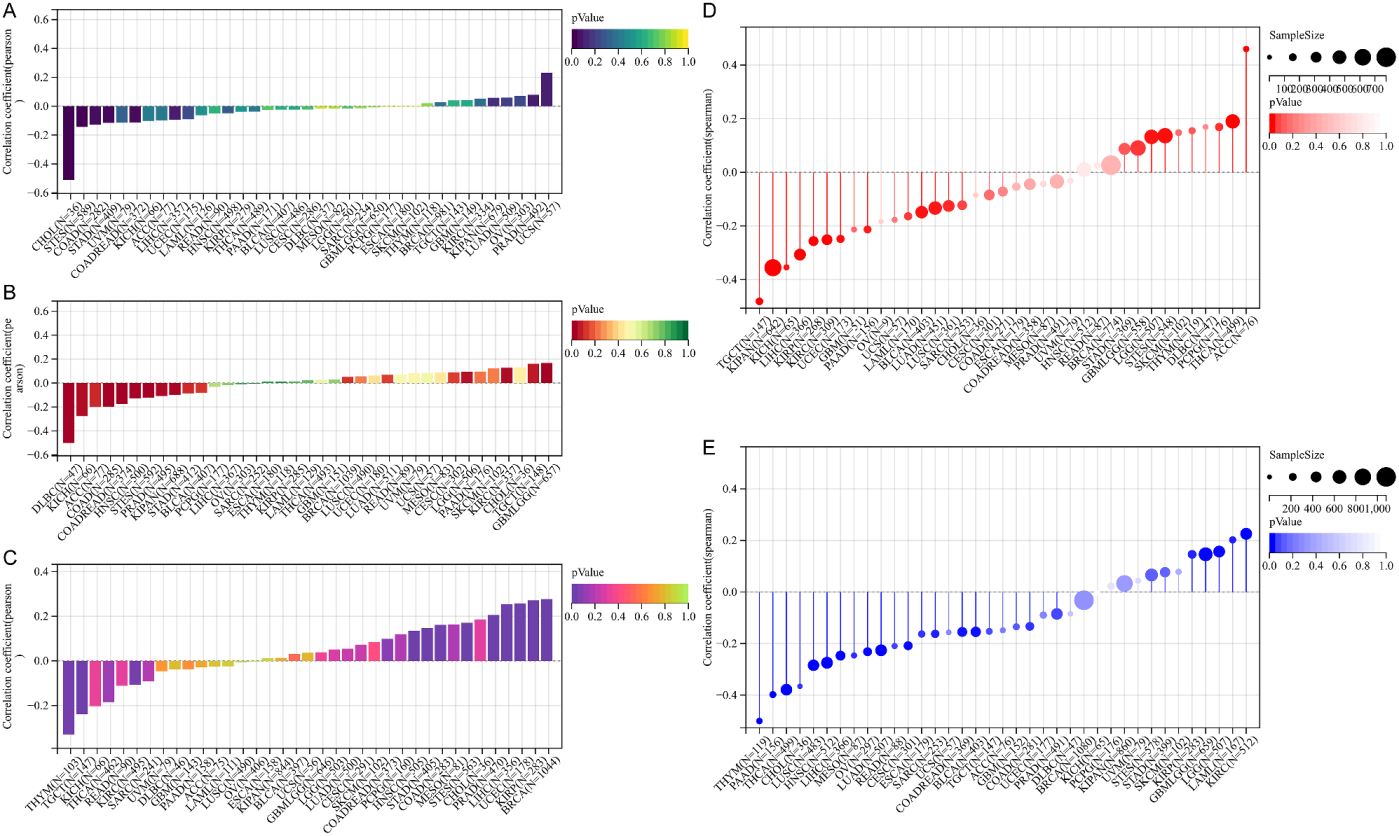
Correlation analysis of glutaminase (GLS) expression and tumor heterogeneity and stemness in pan-cancer. (**A**) Correlation between GLS expression and TMB; (**B**) Correlation between GLS expression and MSI; (**C**) Correlation between GLS expression and HRD; (**D**) Correlation between GLS expression and DNAss; (**E**) Correlation between GLS expression and RNAss; TMB, tumor mutational burden; MSI, microsatellite instability; HRD, homologous recombination deficiency; DNAss, DNA methylation-based stemness score; RNAss, RNA expression-based stemness score. GLS, glutaminase. *P < 0.05, **P < 0.01, ***P < 0.001

By calculating the Pearson’s correlation coefficient in each type of cancers, GLS expression was observed to be negatively related to the TMB in COAD (*r* = -0.13, *p* = 0.03), COAD&READ (*r* = -0.11, *p* = 0.03), stomach and esophageal carcinoma (STEC) (*r* = -0.14, *p* < 0.001), STAD (*r* = -0.12, *p* = 0.02) and CHOL (*r* = -0.51, *p* = 0.002) (Fig. 18A). For MSI, significant associations in 11 cancers were obtained, including the positive correlation in GBM&LGG (*r* = 0.17, *p* < 0.001), LGG (*r* = 0.09, *p* = 0.04) and KIRC (*r* = 0.13, *p* = 0.02), and the negative correlation in COAD (*r* = -0.20, *p* < 0.001), COAD&READ (*r* = -0.17, *p* < 0.001), STEC (*r* = -0.12, *p* = 0.003), pan-kidney cohort (KICH + KIRC + KIRP) (KIPAN) (*r* = -0.10, *p* = 0.01), PRAD (*r* = -0.11, *p* = 0.02), HNSC (*r* = -0.13, *p* = 0.004), KICH (*r* = -0.28, *p* = 0.02) and DLBC (*r* = -0.50, *p* < 0.001) (Fig. 18B). As to HRD, GLS expression was positively associated in COAD (*r* = 0.16, *p* = 0.001), BRCA (*r* = 0.28, *p* = 2.1e-19), STEC (*r* = 0.17, *p* < 0.001), KIRP (*r* = 0.27, *p* = 0.002), STAD (*r* = 0.15, *p* = 0.004), PRAD (*r* = 0.21, *p* < 0.001), UCEC (*r* = 0.26, *p* = 0.002), HNSC (*r* = 0.14, *p* = 0.003) and LIHC (*r* = 0.25, *p* < 0.001), while negatively associated in KIRC (*r* = -0.11, *p* = 0.02), THYM (*r* = -0.33, *p* = 0.02) and TGCT (*r* = -0.24, *p* = 0.004) (Fig. 18C).

We further explored the correlation between GLS expression and tumor stemness score in pan-cancer using the Spearman method. In short, GLS expression was positively related to the DNAss in GBM&LGG (*r* = 0.09, *p* = 0.03), LGG (*r* = 0.13, *p* = 0.003), STEC (*r* = 0.14, *p* = 0.001), THCA (*r* = 0.19, *p* < 0.001), PCPG (*r* = 0.17, *p* = 0.02) and ACC (*r* = 0.46, *p* < 0.001), whereas the negative correlation was observed in LUAD (*r* = -0.13, *p* = 0.005), AML (*r* = -0.16, *p* = 0.03), KIRP (*r* = -0.26, *p* < 0.001), KIPAN (*r* = -0.36, *p* = 1.4e-20), UCEC (*r* = -0.25, *p* < 0.001), KIRC (*r* = -0.25, *p* < 0.001), LUSC (*r* = -0.13, *p* = 0.02), LIHC (*r* = -0.31, *p* = 2.0e-9), PAAD (*r* = -0.21, *p* = 0.008), TGCT (*r* = -0.48, *p* = 6.9e-10), BLCA (*r* = -0.15, *p* = 0.003) and KICH (*r* = -0.35, *p* = 0.004) (Fig. 18D). For RNAss, the positive correlation was observed in GBM&LGG (*r* = 0.15, *p* < 0.001), LGG (*r* = 0.16, *p* < 0.001), AML (*r* = 0.20, *p* = 0.009), KIRP (*r* = 0.15, *p* = 0.01) and KIRC (*r* = 0.23, *p* = 2.4e-7), while the opposite was in CESC (*r* = -0.21, *p* < 0.001), LUAD (*r* = -0.23, *p* = 2.7e-7), COAD (*r* = -0.13, *p* = 0.03), COAD&READ (*r* = -0.15, *p* = 0.003), ESCA (*r* = -0.16, *p* = 0.03), SARC (*r* = -0.16, *p* = 0.01), HNSC (*r* = -0.27, *p* = 2.6e-10), LUSC (*r* = -0.28, *p* = 2.00e-10), THYM (*r* = -0.50, *p* = 6.7e-9), LIHC (*r* = -0.25, *p* < 0.001), THCA (*r* = -0.38, *p* = 1.8e-18), MESO (*r* = -0.25, *p* = 0.02), PAAD (*r* = -0.40, *p* = 3.4e-7), OV (*r* = -0.23, *p* < 0.001), BLCA (*r* = -0.15, *p* = 0.002) and CHOL (*r* = -0.37, *p* = 0.03) (Fig. 18E).

### Correlation of glutaminase (GLS) with tumor-immune infiltration

**Fig. 19:**
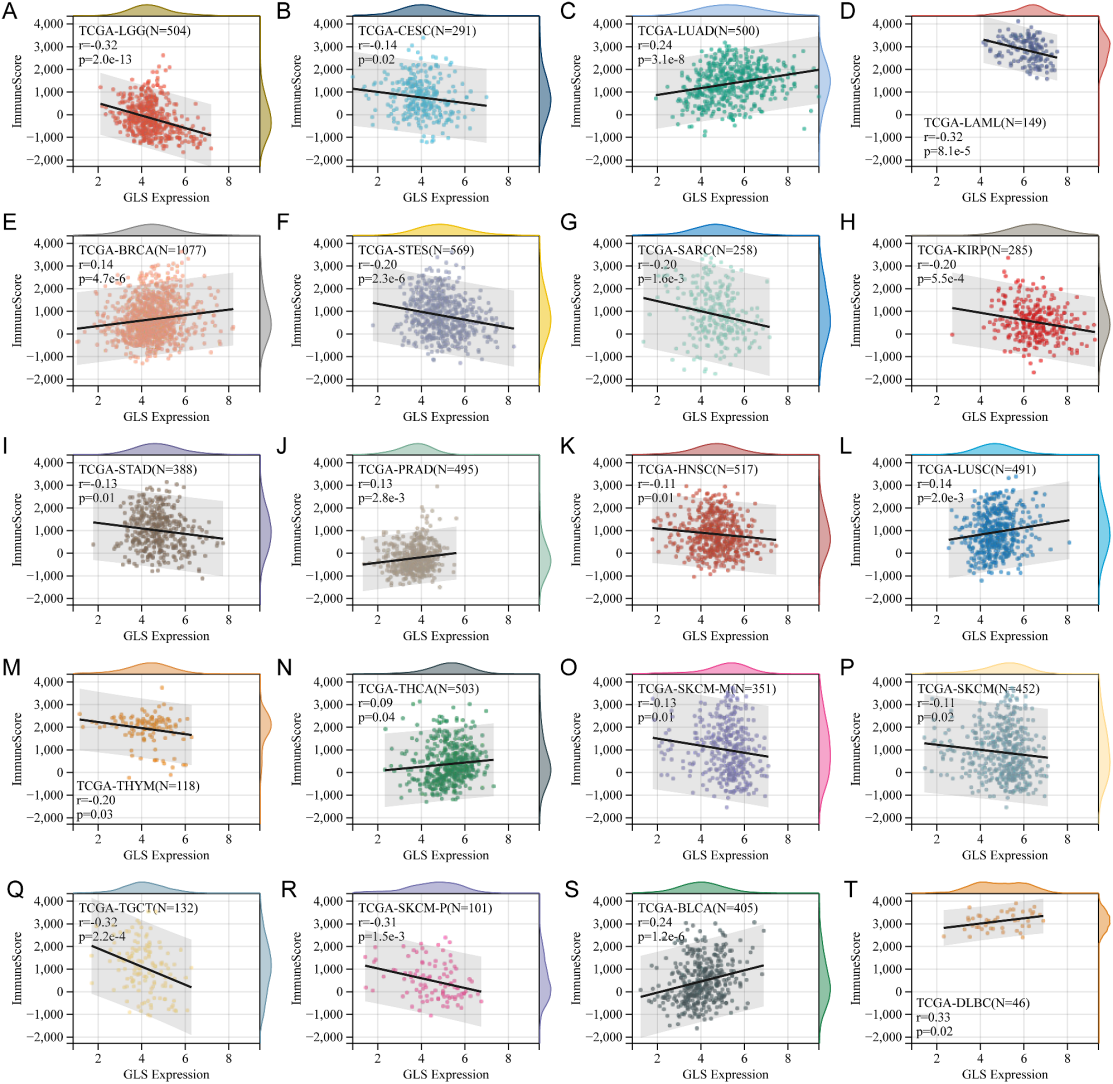
Correlation analysis of glutaminase (GLS) expression and tumor immune infiltration in pan-cancer. (**A**) LGG; (**B**) CESE; (**C**) LUAD; (**D**) AML; (**E**) BRCA; (**F**) STEC; (**G**) SARC; (**H**) KIRP; (**I**) STAD; (**J**) PRAD; (**K**) HNSC; (**L**) LUSC; (**M**) THYM; (**N**) THCA; (**O**) SKCM-M; (**P**) SKCM; (**Q**) TGCT; (**R**) SKCM-P; (**S**) BLCA; (**T**) DLBC. *P < 0.05, **P < 0.01, ***P < 0.001

Besides the immune cell infiltration analysis above, the correlation between GLS expression and overall tumor-immune infiltration in pan-cancer was further estimated using the “immune score”. The results demonstrated that GLS had a positive association with an immune score in 7 cancers, including LUAD (*r* = 0.24, *p* = 3.1e-8) (Fig. 19C), BRCA (*r* = 0.14, *p* = 4.7e-6) (Fig. 19E), PRAD (*r* = 0.13, *p* = 2.8e-3) (Fig. 19J), LUSC (*r* = 0.14, *p* = 2.0e-3) (Fig. 19L), THCA (*r* = 0.09, *p* = 0.04) (Fig. 19N), BLCA (*r* = 0.24, *p* = 1.2e-6) (Fig. 19S) and DLBC (*r* = 0.33, *p* = 0.02) (Fig. 19T). In comparison, the negative association was identified in 13 cancers, including LGG (*r* = -0.32, *p* = 2.0e-13) (Fig. 19A), CESC (*r* = -0.14, *p* = 0.02) (Fig. 19B), AML (*r* = -0.32, *p* = 8.1e-5) (Fig. 19D), STEC (*r* = -0.20, *p* = 2.3e-6) (Fig. 19F), SARC (*r* = -0.20, *p* = 1.6e-3) (Fig. 19G), KIRP (*r* = -0.20, *p* = 5.5e-4) (Fig. 19H), STAD (*r* = -0.13, *p* = 0.01) (Fig. 19I), HNSC (*r* = -0.11, *p* = 0.01) (Fig. 19K), THYM (*r* = -0.20, *p* = 0.03) (Fig. 19M), SKCM-metastasis (SKCM-M) (*r* = -0.13, *p* = 0.01) (Fig. 19O), SKCM (*r* = -0.11, *p* = 0.02) (Fig. 19P), TGCT (*r* = -0.32, *p* = 2.2e-4) (Fig. 19Q) and SKCM-P (*r* = -0.31, *p* = 1.5e-3) (Fig. 19R).

## Glutaminase (GLS)-related mutation landscape and drug sensitivity

**Fig. 20:**
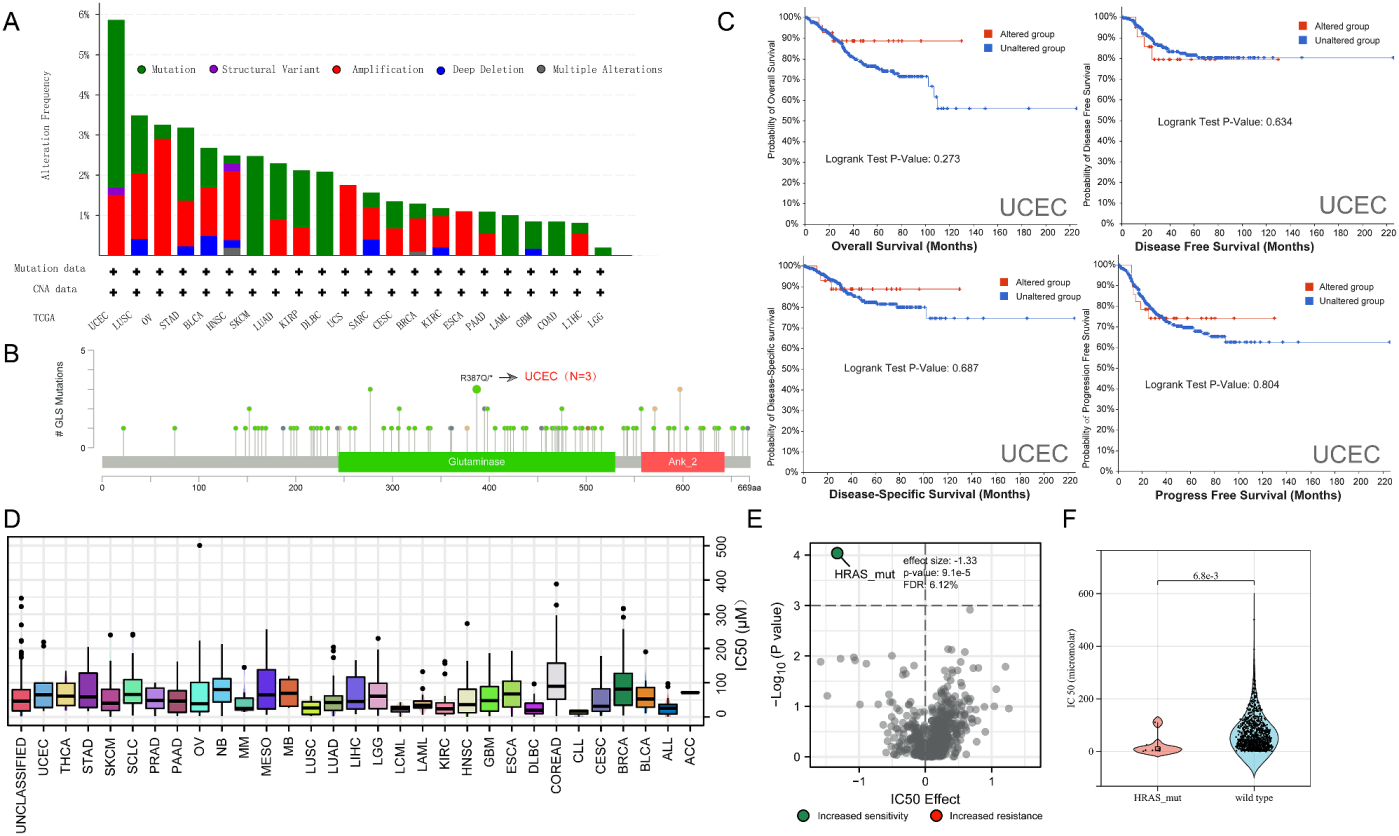
Genetic variation features of glutaminase (GLS) in pan-cancer via cBioPortal and drug sensitivity analysis. (**A**) The mutation frequency and types in diverse cancers; (**B**) Mutation site with the highest frequency (R387Q/*) of GLS and related carcinomas; (**C**) GLS alteration impact on OS, DSS, DFS, and PFS of UCEC; (**D**) IC50 distribution of GLS-related drug for by cancer type; (**E**) Correlation between gene mutation type and GLS-related drug sensitivity; (**F**) Drug responses based on GLS specific mutations. GLS, glutaminase; OS, overall survival; DSS, disease-specific survival; DFS, disease free survival; PFS, progression free survival; UCEC, uterine corpus endometrial carcinoma; IC50, Inhibitory concentration 50

The GLS-related genetic variation landscape across different carcinomas was searched from the TCGA database. The highest alteration frequency of GLS (–6%) appeared in UCEC with “mutation” as the primary type (Fig. 20A). As the detailed information shown in Fig. 20B, R387Q/* alteration in the Glutaminase domain was the most common mutation site, which was detected in three cases of UCEC. This change might lead to a frameshift at the 387 site of GLS protein with translation from arginine (R) to glutamine (Q). Moreover, we explored the relationship between GLS mutation and prognosis in UCEC patients. Although the patients in the unaltered group seemed to have a worse prognosis, there were no significant differences been observed in OS, DFS, DSS and PFS (all *p* > 0.05) (Fig. 20C).

BPTES, the unique drug predicted to target GLS, was designated for experimental use only. We subsequently estimated the IC50 distribution of BPTES in pan-cancer and found a wide range of IC50 differences in different tumor tissues (Fig. 20D). On the other hand, by assessing the correlation between gene mutation and drug sensitivity, we observed that HRAS mutation might increase the BPTES sensitivity with an effect size of -1.33 (*p* = 9.1e-5) (Fig. 20E). Furthermore, there was a significant difference between HRAS mutation group and wild type group (*p* = 6.8e-3) (Fig. 20F), and details were listed in Table 3.

**Fig. 21:**
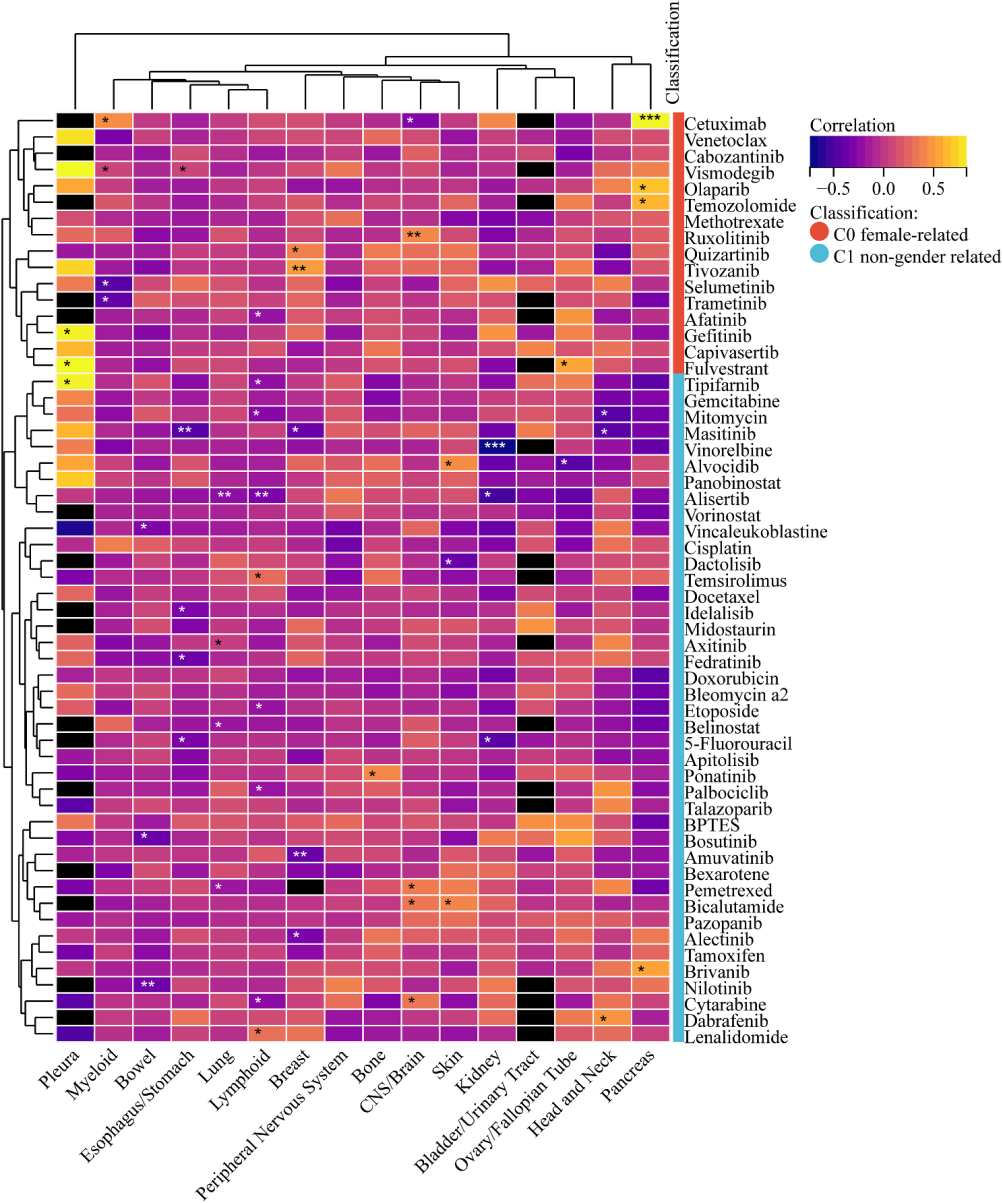
Prediction and correlation analysis of glutaminase (GLS) associated drugs. Sensitivity heatmap of common drugs targeting glutaminase (GLS) in pan-cancer

Given the lack of effective targeting drugs for GLS in clinic, we assessed the correlation between 57 clinically common anti-tumor drugs and GLS in pan-cancer. The results were summarized in Fig. 21. Shortly specking, we observed the most significant positive correlation of cetuximab with GLS in pancreatic cancer (*r* = 0.82, *p* < 0.001), while the most significant negative correlation of vinorelbine with GLS in kidney cancers (*r* = -0.74, *p* < 0.001).

**Table 3:** BPTES IC50 values for HRAS_mut on pan-cancer

	HRAS_mut	Wild type	MWW p-value
Number of cell lines	7	876	
Median	4.9117	44.778	
Geometric mean	7.9043	38.273	
ALL groups			**0.00680**
ALL	0	23	-
ACC	0	1	-
BLCA	1	17	0.11111
BRCA	1	47	0.27892
CESC	0	12	-
CLL	0	2	-
COREAD	0	45	-
DLBC	0	29	-
ESCA	0	35	-
GBM	0	32	-
HNSC	1	38	0.11997
KIRC	0	29	-
LIHC	0	15	-
LUAD	0	60	-
LUSC	1	13	0.57143
LCML	0	10	-
LAML	0	23	-
LGG	0	16	-
MESO	0	17	-
MB	0	4	-
MM	0	15	-
NB	0	24	-
OV	0	32	-
PAAD	0	27	-
PRAD	0	5	-
SKCM	1	52	0.10924
STAD	0	22	-
SCLC	0	51	-
THCA	0	13	-
UCEC	0	9	-
UNCLASSIFIED	2	158	0.89614

*Compared with each group (Wilcoxon rank sum test). *P* < 0.05 means statistically significant (highlighted in bold)

## Discussion

GLS, as one of the genetic regulators of the pyruvate dehydrogenase (PDH) complex, plays a critical role in the rate-limiting step in mitochondrial pyruvate decarboxylation, which links the TCA cycle to glycolysis and fat and amino acid metabolism [63]. Current research suggests that cancer cells, especially those with an uncontrolled expression of oncogenes/onco-suppressor genes, rely significantly on the metabolic reprogramming of the TCA cycle (Warburg effect) to produce energy and promote tumor progression [64]. Although the mechanism of tumor metabolic reprogramming is complex and remains to be elucidated, Tsvetkov revealed a novel cell death pattern, “cuproptosis”, involving mitochondrial metabolism [11]. This finding suggests new metabolic-related targets for anti-tumor therapy. Hence, as one of the key molecules in the copper-induced cell death pathway, GLS has been endowed with a new anti-tumor mechanism and function. Previous studies reported that GLS exerted essential functions in tumor proliferation [65] and metastasis [66]; however, no definitive research has comprehensively appraised the importance of GLS in pan-cancer. Our study is a first attempt to explore and confirm the oncogenic role of GLS in pan-cancer using the bioinformatic method based on the multi-omics scale.

Using the TCGA, GTEx, and CCLE databases, we comprehensively evaluated the GLS expression level across different cancers. Briefly, GLS expression level was down-regulated significantly in 17 human carcinomas and up-regulated in 11 other cancers. This might indicate that GLS serves as an onco-suppressor gene in most malignancies, and the approach of energy metabolism in these tumors might have changed. In this research, however, GLS protein expression was lower in PAAD and higher in LUAD, which was inconsistent with its expression at the transcriptional level. This finding presumably suggested that one or more unknown mechanisms took part in the translation process of GLS in PAAD and LUAD. As previous studies reported, GLS was expressed highly in PAAD and could be regulated by transcription factor EB (TFEB) [67] and SUCLA2 ^23^, which suggested that there might exist a complex regulation network related to GLS in PAAD. All in all, these findings provided a direction for further exploration.

In addition, the analysis outcomes of this paper revealed that GLS expression was significantly correlated with different molecular subtypes in 11 cancers and different immune subtypes in 12 cancers (Figs. 4 and 5). The differential expression in distinct subtypes of specific tumor types might provide new and meaningful entry points for further exploration of the oncogenic role of GLS. Then, we were surprised to discovered that GLS had an outstanding diagnostic value in 19 cancer types, especially in predicting CHOL, OV and UCS with extremely high accuracy (AUC > 0.9) (Fig. 6). Also, GLS was observed to be significantly associated with the OS, DSS and PFI in KIRC, LGG and UCEC, which indicated that it might be a remarkable prognostic indicator for above cancers. These results initially demonstrated the excellent diagnostic and prognostic capability of GLS in pan-cancer, suggesting that it may act as a promising biomarker for precision oncology. To be consistent with our knowledge and prediction, the bio-function of GLS searched from the GO and KEGG enrichment analysis was involved in the amino acid metabolic process, oxidoreductase activity, acting the aldehyde or oxo group of donors and alanine, aspartate and glutamate metabolism (Fig. 8). It is worth highlighting that GLS is not only critical for the intra-cellular metabolic process but also essential for the connection of cell-cell energy transfer and metabolism.

Furthermore, UCEC was considered the most significant tumor type correlated with GLS according to the comprehensive analyses. Therefore, we emphasized the exploration of the clinical correlation of GLS with UCEC patients, including race, surgical resection, radiation therapy, primary therapy outcome, clinical stage, weight, height, BMI and histological type/grade. Although GLS was expressed lower in UCEC than in corresponding normal tissues, UCEC patients with increased GLS expression had worse outcomes for OS, DSS and PFI, as did patients in a diverse set of clinical subgroups. We speculated that elevated expression of GLS might induce changes in some biomarkers or pathways not known yet, leading to a worse prognosis for UCEC patients; and metabolism reprogramming and activation of oncogenic pathways were the most possible underlying causes. However, all conjectures require to be verified by further experiments. Though many questions about GLS remain unresolved, there is no doubt that it is a unique prognostic signature of UCEC, supported by a prior study involved in exploring prognostic signatures in UCEC [68].

More importantly, 5 clinically independent risk factors were further screened through univariate and multivariate regression analysis to establish an innovative GLS-related nomogram. The nomogram analysis results demonstrated a high degree of robustness and accuracy for predicting 1-, 3- and 5-year outcomes of UCEC patients (Fig. 13). This work is of great significance in providing a more in-depth and comprehensive cognition on the clinical correlation between GLS and UCEC. Given the absence of screening programs for early detections of UCEC [69], our findings offered new insights and valuable tool for clinical application. In addition, we also explored GLS-related positive/negative co-expression genes, DEGs between GLS up- and down-regulated groups, and enriched mechanisms and pathways in UCEC. Finally, 12 algorithms were used for screening the hub genes of DEGs. These findings contribute to our understanding of the potential oncogenic role of GLS in UCEC, particularly in relation to cilia motility and neurohormones. Despite requiring further experimental validation, the speculations in this study offer new perspectives for future research on GLS.

Besides, we evaluated the association of GLS with tumor-immune infiltration, genetic heterogeneity, tumor stemness and drug sensitivity in pan-cancer. Five types of immune cells highly correlated were obtained, including Tregs in SKCM-P, monocytes in AML, macrophages_M2 in TGCT, Tregs in UCEC and T_cells_CD4_memory_resting in PRAD (Fig. 17). Jiang et al. [70] revealed the essential role of GLS in macrophage M1 polarization and a possible similar mechanism contributing to the high infiltration of macrophages M2 in TGCT. Zhou et al. stated that up-regulated GLS expression promoted lactate production, thereby influencing Tregs differentiation and resulting in immune evasion [71]. Based on this, we speculate that this mechanism may also exist in UCEC. As for tumor-immune infiltration, the most positive correlation between GLS and the immune score was observed in DLBC, while the most negative correlation was in TGCT and AML (Fig. 19). These findings enlighten us that GLS may play an essential role in tumor-immune interaction, however, the underlying interaction mechanisms necessitate extensive basic experimentation for further exploration and validation. Collectively, it is of great interest and importance to connect the GLS-related cuproptosis and tumor-immune infiltration, which is abundant in the unknown for in-depth exploration.

According to the tumor heterogeneity analysis, we found that GLS expression was most negatively correlated with TMB in CHOL (*r* = -0.51) (Fig. 18A), which indicated that down-regulated GLS might serve as a breakthrough for enhancing immunotherapy in CHOL. Next, GLS expression was observed to be most negatively associated with MSI in DLBC (*r* = -0.50) (Fig. 18B), suggesting that decreased GLS might strengthen the immunogenicity of DLBC to get more benefits from immunotherapy. As for HRD, the most significant negative correlation was disclosed in THYM (*r* = -0.33) (Fig. 18C), which inferred that lower GLS expression could potentially promote the sensitivity of platinum-based chemotherapy and PARP inhibitors to THYM. In addition, the analysis outcomes revealed the most significant positive association between GLS and DNAss in ACC (*r* = 0.46), as well as the most significant negative association of GLS with DNAss in TGCT (*r* = -0.48) and with RNAss in THYM (*r* = -0.50) (Fig. 18). All of these findings indicated that GLS was probably associated with drug resistance, cancer recurrence and tumor proliferation, though these findings needed to be further explored and verified by experiments. Finally, due to a lack of relevant data, we only made a preliminary attempt to evaluate the correlation between GLS-related drug sensitivity and gene alteration. Specifically, HRAS mutation was the most significant mutation type related to IC50 of BPTES, and cetuximab and vinorelbine were the two anti-tumor drugs related to GLS most closely. Despite the limited therapy information, there is ample opportunity for further in-depth research in this field.

Although the oncogenic role of GLS through the Warburg effect had been previously identified, its role within the context of cuproptosis-related pathways in tumors was first proposed in this study. Our research revealed the exceptional diagnostic and prognostic value of GLS across various tumors, particularly in endometrial cancer. Besides, we developed a novel prognostic model for UCEC and verified its accuracy and robustness, offering new hope in a field that has seen few significant breakthroughs. This study provided a comprehensive overview of the mechanisms and implications of GLS in cancer broadly. Moreover, serving as a valuable addition to the existing literature, this study offered guidance for future research directions. Despite our extensive analysis, our study has several notable limitations. Firstly, we used multiple databases and statistics to elaborate on the oncogenic role and clinical correlations of of GLS in pan-cancer while absent clinical datasets from other databases or the real world. Notably, potential confounders and sample size may also contribute to the results biases. Improving the sample homogeneity and collaborating with multiple centers are necessary for providing further evidence on the clinical translation of GLS in UCEC. Besides, reliable verification and clear evidence with high confidence need to be provided by biological experiments in the future. Furthermore, we did not explore the aspect of the tumor-immune microenvironment and drug prediction due to the shortage of available data. Anyhow, the current results are still helpful in lighting the way for future research.

## Conclusion

We created the first pan-cancer bioinformatics landscape of GLS and revealed its diagnostic and prognostic value for UCEC. Furthermore, the discovery of the unique expression patterns of GLS across different cancer types suggested the potential of GLS serving as a promising biomarker for cancer detection and prognosis. Besides, this study identified potential drugs and therapies linked to GLS, marking a significant step toward targeted cancer therapy. These findings not only lay the groundwork for clinical translation of UCEC screening biomarkers but also point towards directions for further mechanistic validation in pan-cancer. Despite lingering mysteries, future breakthroughs need to be sought through integrating clinical multi-center data and in-depth exploration of potential regulatory mechanisms in basic experiments.

### Electronic supplementary material

Below is the link to the electronic supplementary material.

Supplementary Material 1

Supplementary Material 2

## Data Availability

The original contributions presented in the study are included in the article/supplementary material, further inquiries can be directed to the corresponding author/s.All data and original files in our work are freely available under a ‘Creative Commons BY 4.0’ license. All methods were carried out in accordance with relevant guidelines.

## Abbreviations

ACC: Adrenocortical carcinoma
ITH: Intra-tumor heterogeneity
TCA: Tricarboxylic acid
TMB: Tumor mutation burden
GLS: Glutaminase
MSI: Microsatellite instability
UCEC: Uterine corpus endometrial carcinoma
HRD: Homologous recombination deficiency
UCSC: University of California Santa Cruz
DNAss: Methylation-based stemness score
TPM: Transcripts per million
RNAss: RNA-based stemness score
HPA: Human protein atlas
CNA: Copy number alteration
CCLE: Cancer cell line encyclopedia
DFS: Disease-free survival
TCGA: The cancer genome atlas
PFS: Progression-free survival
GTEx: Genotype-tissue expression
IC50: Half-maximal inhibitory concentration
GEPIA: Gene expression profiling interactive analysis
UALCAN: The University of Alabama at Birmingham Cancer
GDSC: Genomics of Drug Sensitivity in Cancer
WT: Wild type
CPTAC: Clinical proteomic tumor analysis consortium
COAD: Colon adenocarcinoma
TISIDB: Tumor-immune system interaction database
HNSC: head and neck squamous cell carcinoma
ROC: Receiver operating characteristic
LIHC: Liver hepatocellular carcinoma
AUC: Area under the curve
CHOL: Cholangiocarcinoma
K-M: Kaplan-Meier
OS: Overall survival
ESCA: Esophageal carcinoma
STAD: Stomach adenocarcinoma
DSS: Disease-specific survival
BRCA: Breast invasive carcinoma
PFI: Progression-free interval
KIRC: Kidney renal clear cell carcinoma
HR: Hazard ratio
KICH: Kidney chromophobe
CI: Confidence interval
LUSC: Lung squamous cell carcinoma
PPI: Protein-protein interaction
PRAD: Prostate adenocarcinoma
KEGG: Kyoto encyclopedia of genes and genomes
DLBC: Diffuse large B cell lymphoma
GO: Gene ontology
AML: Acute myeloid leukemia
DCA: Decision curve analysis
PAAD: Pancreatic adenocarcinoma
FC: Fold-change
READ: Rectal adenocarcinoma
DEGs: Differential expressed genes
THYM: Thymoma
BP: Biological process
GBM: Glioblastoma multiforme
CC: Cellular component
LGG: Brain low-grade glioma
MF: Molecular function
LUAD: Lung adenocarcinoma
CESC: Cervical squamous cell carcinoma and endocervical adenocarcinoma
OV: Ovarian serous cystadenocarcinoma
SKCM: Skin cutaneous melanoma
THCA: Thyroid carcinoma
UCS: Uterine carcinosarcoma
Tregs: T cells regulatory
PCPG: Pheochromocytoma and paraganglioma
BLCA: Bladder urothelial carcinoma
TCGA-SKCM-P: TCGA-SKCM-primary
STEC: Stomach and esophageal carcinoma
KIPAN: pan-kidney cohort (KICH + KIRC + KIRP)
SKCM-M: SKCM-metastasis
PDH: pyruvate dehydrogenase
TFEB: transcription factor EB
