# Supplementary figures and images for: A paradigm shift in cancer research based on integrative multi-omics approaches: glutaminase serves as a pioneering cuproptosis-related gene in pan-cancer
Source: BMC Womens Health. 2024 Apr 2;24:213. doi: 10.1186/s12905-024-03061-8 (PMC10988933; doi:10.1186/s12905-024-03061-8)

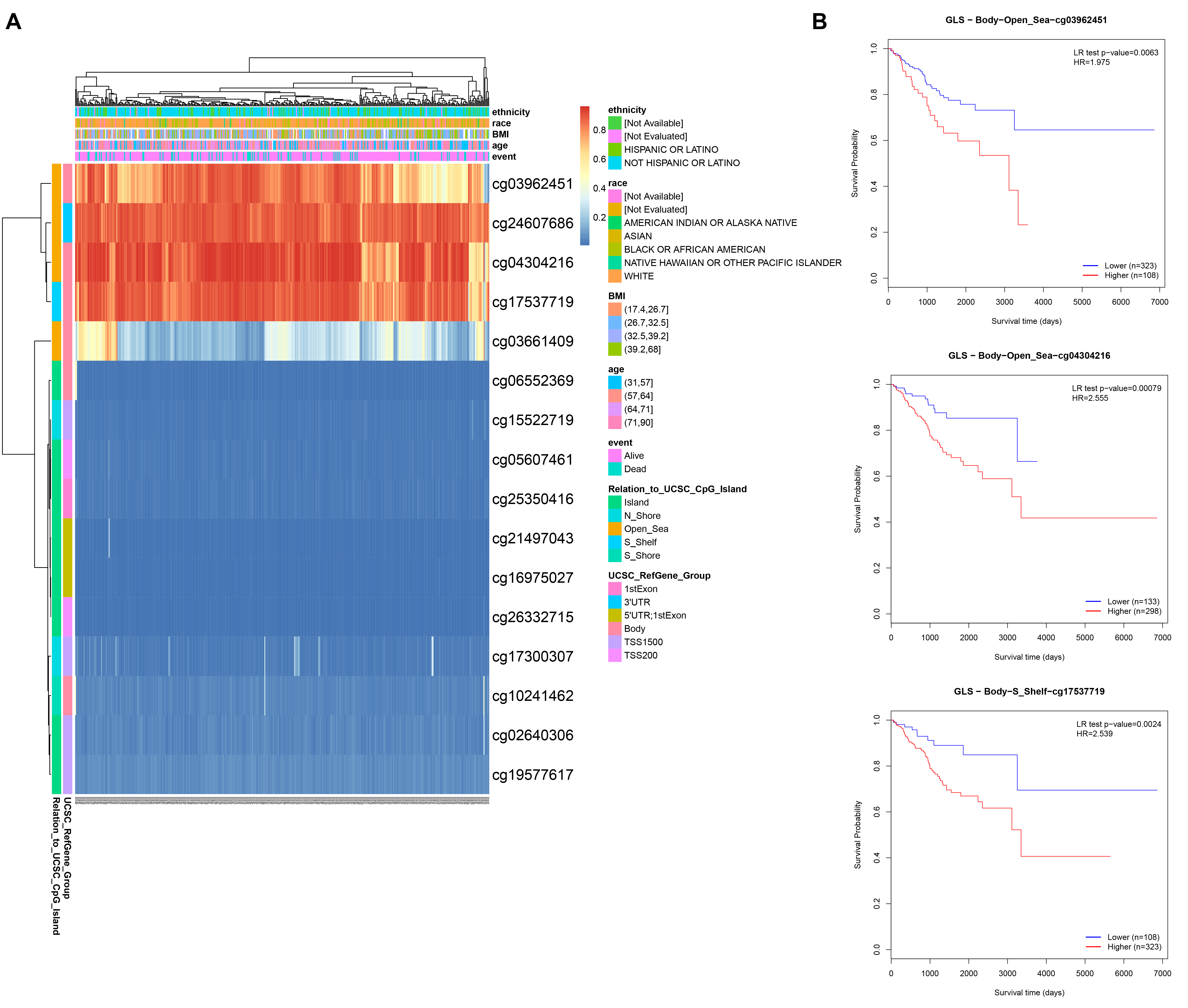

Supplement: Supplementary file 1 — Supplementary Material 1 [file 12905_2024_3061_MOESM1_ESM.tif]

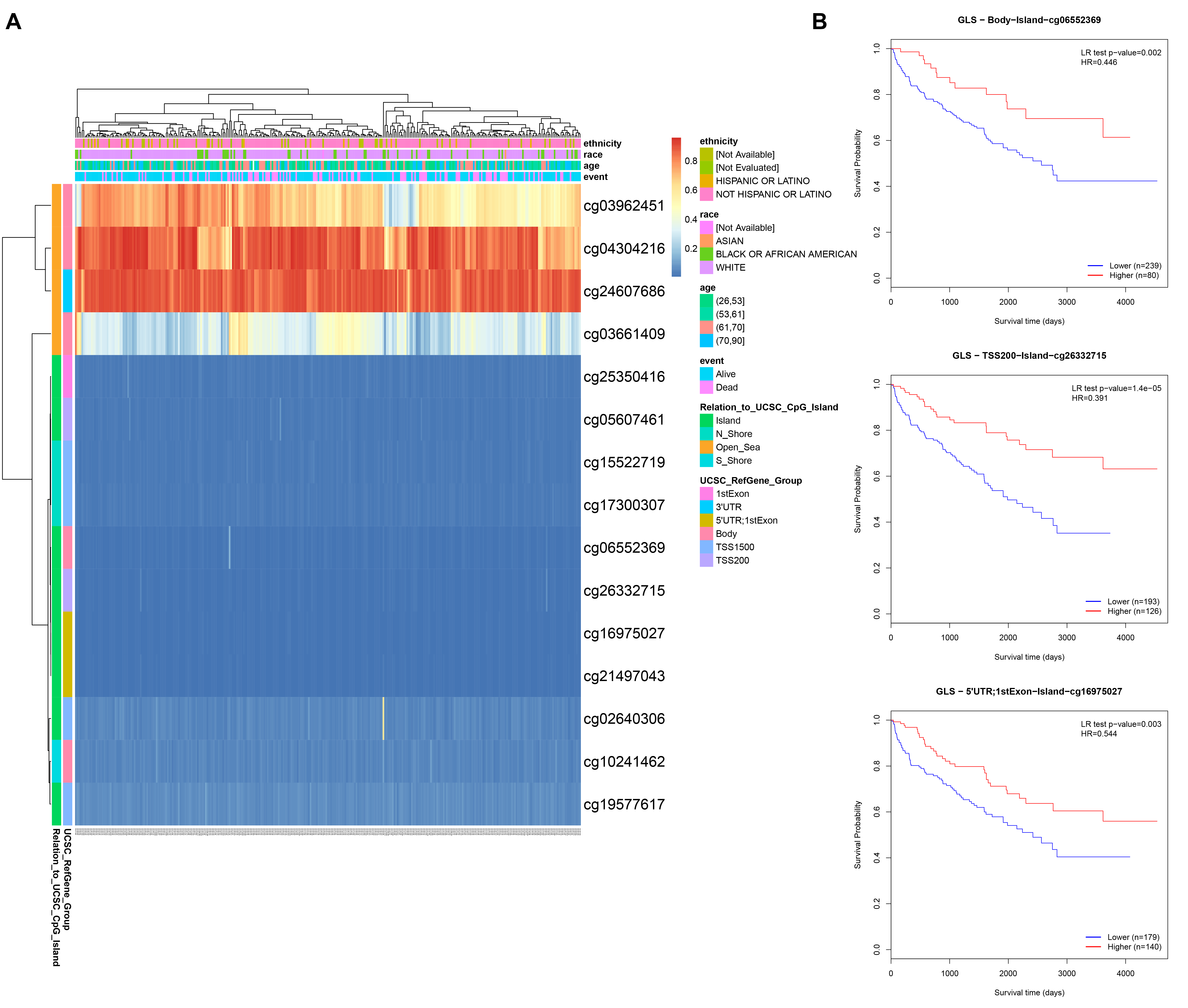

Supplement: Supplementary file 2 — Supplementary Material 2 [file 12905_2024_3061_MOESM2_ESM.tif]
